# 1,2,3-Triazoles: Controlled Switches in Logic Gate Applications

**DOI:** 10.3390/s23157000

**Published:** 2023-08-07

**Authors:** Debanjana Ghosh, Austin Atkinson, Jaclyn Gibson, Harini Subbaiahgari, Weihua Ming, Clifford Padgett, Karelle S. Aiken, Shainaz M. Landge

**Affiliations:** 1 Department of Chemistry and Biochemistry, Georgia Southern University (Statesboro Campus), 521 College of Education Drive, Statesboro, GA 30460-8064, USA; dghosh@siue.edu (D.G.);; 2 Department of Chemistry, Science Building West, Southern Illinois University Edwardsville, Edwardsville, IL 62026-1652, USA; 3 Department of Chemistry and Biochemistry, Georgia Southern University (Armstrong Campus), 11935 Abercorn Street, Savannah, GA 31419, USA

**Keywords:** 1,2,3-triazole, click chemistry, PTP, fluorescence sensor, dual sensing, logic gate, switches

## Abstract

A 1,2,3-triazole-based chemosensor is used for selective switching in logic gate operations through colorimetric and fluorometric response mechanisms. The molecular probe synthesized via “click chemistry” resulted in a non-fluorescent 1,4-diaryl-1,2,3-triazole with a phenol moiety (**PTP**). However, upon sensing fluoride, it TURNS ON the molecule’s fluorescence. The TURN-OFF order occurs through fluorescence quenching of the sensor when metal ions, e.g., Cu^2+^, and Zn^2+^, are added to the **PTP**-fluoride ensemble. A detailed characterization using Nuclear Magnetic Resonance (NMR) spectroscopy in a sequential titration study substantiated the photophysical characteristics of **PTP** through UV-Vis absorption and fluorescence profiles. A combination of fluorescence OFF-ON-OFF sequences provides evidence of 1,2,3-triazoles being controlled switches applicable to multimodal logic operations. The “INH” gate was constructed based on the fluorescence output of **PTP** when the inputs are F^−^ and Zn^2+^. The “IMP” and “OR” gates were created on the colorimetric output responses using the probe’s absorption with multiple inputs (F^−^ and Zn^2+^ or Cu^2+^). The **PTP** sensor is the best example of the “Write-Read-Erase-Read” mimic.

## 1. Introduction

Bringing the macroscopic properties to the molecular level is a stepping stone in molecular and supramolecular chemistry [1]. For instance, the miniaturization of digital devices is reaching its limits in storage capacity, size, retaining of information, and processing speed [2,3]. Small, inexpensive organic molecular sensors can easily be utilized to create logic gates, switches, keypad locks, security systems, and memory machines by manipulating the core design based on the desired function [4].

Molecular logic gate operations occur with a specific sequence of events in which one or more inputs (e.g., chemical, electrical, or optical) are converted into a single output (e.g., chemical, electrical, or optical) [5,6,7,8]. Since the input–output interchange is occurring at the molecular level, these logic gates are in essence the building blocks of nano-devices. Typically, a single molecule is an information processing unit [9] operating “physically wire-free”; it can be used as a portable tool [10]. Their implementation in computing can lead to a higher order of speed that is impossible to achieve with conventional electronic devices [11]. For instance, at any one time, a molecule can perform multiple operations based on different outputs that result from a particular sequence of events [10].

Logic gates, in particular, use the Boolean function for the basic physical construct of digital devices. The binary inputs (combination of “0” or “1”) are translated into another single binary output (“0” or “1”), where “0” represents low/NO/FALSE, and “1” represents high/YES/TRUE. Generally, molecular logic gates operate wirelessly to process chemical (pH, cations, anions) inputs and generate analytical responses (colorimetric or fluorescent signals) as their outputs [12]. Most logic gates have two or multiple inputs and one output that can be adapted at a molecular level. The greatest challenge for chemists is creating stable, fast, efficient, and reversible (resettable) molecular logic systems [7]. Optically and chemically stable triazole probes, with straightforward green syntheses, targeted design, and sensitive and selective ion-recognition properties, can bridge that gap. Furthermore, the ability to structurally tune the signal output to a specific range of wavelengths for targeted applications perfectly ties into the goals of the logic gate investigations.

In this study, we will exploit the recognition capabilities of the 1,2,3-triazole chemosensors [13] in logic applications to access real-time diagnostic and memory devices [14]. The triazole derivatives synthesized by our group are known for sensing and reversible characteristics [15,16,17,18,19,20]. We realized that logic functions can be aptly used to access applications such as real-time diagnostics, molecular devices, and therapeutics [14,21]. The synthesized triazole sensors’ cation and anion recognition can act as “inputs” to show fluorescence or color change signals as “output”. The “ON” process in the logic gate will be monitored via a fluorometric or colorimetric response that is displayed when the probe binds to anions or cations. The “OFF” sequence is displayed by the fluorescence quenching/reversal of the original color change by adding metal cations. Based on the ON-OFF functions of chemical inputs, our well-designed chemoreceptors can be used as complementary INHIBIT (INH) and IMPLICATION (IMP) gates, as well as basic OR (IMP/INH/OR) logic functions [22,23]. The INHIBIT gate, a combination of NOT and AND gates, deserves attention because of its non-commutative behavior. This means that the output signal is inhibited by one power input [24]. The IMPLICATION logic operation is performed as a plausible combination of AND, OR, and NOT gates [25]. Other possible routes through intramolecular charge transfer (ICT), such as electron transfer (ET) [26] interactions with fluoride anion, have been used to generate combinational logic gates [27].

Through collaborative efforts in published works, we demonstrated that triazoles appended with ortho-hydroxy phenolic groups can be an efficient anion sensor with a 1:1 binding stoichiometry. The molecule’s fluorescence TURNS ON in the presence of tetrabutylammonium (TBA) salts of fluoride (F^−^) [15]. Cu^2+^ showed a color change under ambient light for metal ion sensing, with a concurrent TURN-OFF fluorescence [16,19]. The stoichiometric ratio depends on the triazole derivative; it is 2:1, sensor: Cu^2+^ with a bis-triazole sensor (BPT) [16] and 1:1 between a phenanthrene triazole (PhTP) and copper (II) [19]. In another work by our group, we established a dual-sensing by 1,2,3 triazole molecules. The molecule showed a change in fluorescence output with F^−^ under the UV lamp; however, a complete quenching of fluorescence was observed with Cu^2+^ [19]. Furthermore, we functionalized the signaling unit with the amino (electron donating) groups and realized the amino (–NH_2_) groups [20] with the core *o*-phenolic aromatic structure displayed efficient signaling and higher selectivity towards F^−^ compared to dihydrogenphosphate (H_2_PO_4_^−^), and acetate (AcO^−^). Having these multiple affirmative outcomes in hand, our hypothesis of building a logic gate is the right path toward future applications.

**Figure 1:**
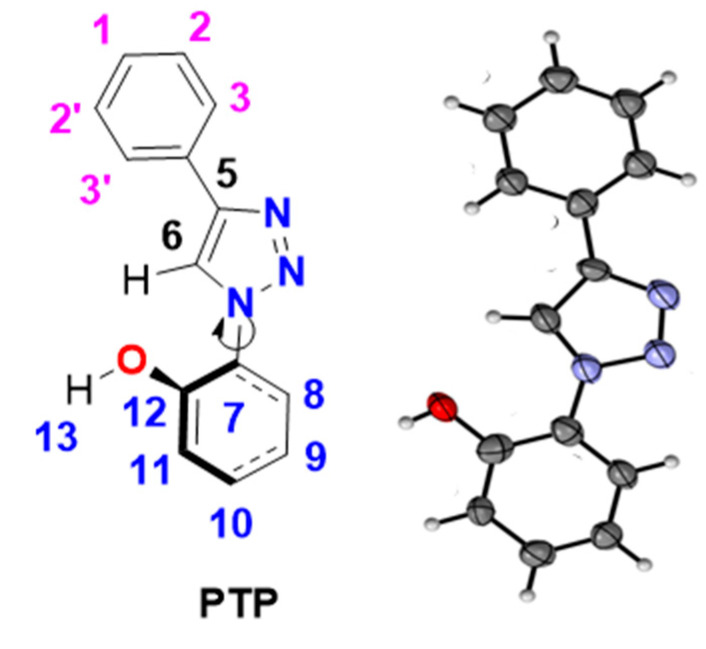
A view of the molecular structure of **PTP**, displacement ellipsoids are drawn at the 50% probability level.

Herein, we report the photophysical characterization of **PTP** (Figure 1) in the presence of anions and cations for the sequential detection of F^−^ and Zn^2+^ or Cu^2+^ through the ON-OFF-ON fluorescence response. Interestingly, the probe has effectively developed multi-modal sequences with IMP, INH, and OR logic gates. The elaborated NMR studies complemented the combinatorial gates constructed. Through this design of multiple logic gates, we aim to achieve a higher level of accuracy in ion recognition and contribute to the arena of developing smart systems [28].

## 2. Experimental Section

### 2.1. Materials and Methods

All chemicals and reactants were obtained from commercial sources and used without further purification. For anion sensing, tetrabutylammonium (TBA) salts of fluoride were used, whereas for recognition of cations, perchlorate salts of Ag^+^, Al^3+^, Cd^2+^, Co^2+^, Cr^3+^, Cu^2+^, Fe^2+^, Fe^3+^, Li^+^, Mg^2+^, Ni^2+,^ and Zn^2+^ were used as received.

Room temperature UV-Vis absorption and steady-state fluorescence measurements were performed using a Shimadzu UV-2450 spectrophotometer (USA) and PerkinElmer LS55 fluorimeter (USA), respectively. The concentration of 1,2,3-triazole was kept close to the order of 10^−5^ mol/L (varies between 1 × 10^−5^ and 5 × 10^−5^) in acetonitrile to avoid any possible intermolecular effect. The stock concentrations for tetrabutylammonium salts of the anions and the perchlorate salts of metal ions were prepared as 1 × 10^−2^ mol/L in acetonitrile. The solvents used were of HPLC grade for the experiment. From all the previous studies from our group with 1,2,3-triazole compounds, acetonitrile was the solvent of choice due to the solubility of the compound.

NMR spectra were recorded on an Agilent MR400DD2 spectrometer with a multinuclear probe with two RF channels and variable temperature capability. ^1^H NMR: 400 MHz and ^13^C NMR: 100 MHz; the solvent used was CD_3_CN and DMSO-*d6* purchased from Sigma-Aldrich. The NMR signals are reported in parts per million (ppm) relative to the residual in the solvent. Signals are described with multiplicity, singlet (s), doublet (d), triplet (t), triplet of doublet (td), quartet (q), and multiplet (m); coupling constants (*J*; Hz) and integration. Melting points were measured with Vernier Melt Station using Vernier LabQuest 2 and are uncorrected. The High-Resolution MS (HRMS) analyses were performed using MALDI, Q-TOF micro, 3200 API, LCMS, and GCMS EI (DI). All the experiments were performed at ambient temperature (27 °C) with air-equilibrated solutions.

### 2.2. Synthetic Procedure

The **PTP** probe was synthesized using the click chemistry approach; 2-Azidophenol [29] and phenylacetylene were used as a starting material with 1:1 equivalent in the tert-butanol/water solvent system. A solution of copper (II) sulfate pentahydrate was added dropwise to the vigorously stirred mixture, followed by sodium ascorbate, refluxing for 24 h. After the reaction, the work-up procedure resulted in a solid product purified by flash column chromatography. The detailed procedure of **PTP** was published elsewhere [15]. Detailed characterization is provided in the supporting information (Supplementary Scheme S1, Figures S1 and S2). 

To understand the structure of the sensor, **PTP** crystals were grown for single-crystal X-ray analysis by slow evaporation of dichloromethane at room temperature. The X-ray structure data were collected at 100 K on a Rigaku XtaLAB Synergy-i XRD diffractometer equipped with Cu-Ka radiation (1.54184 nm) and processed using CrysAlis Pro (Rigaku, Tokyo, Japan) software. The structure was solved using direct methods and refined using full matrix least squares refinement using SHELXT and SHELXL in the Olex2 software package. All non-hydrogen atoms were refined anisotropically. H-atoms were placed in calculated positions and refined with riding model approximations; the structure was resolved to (R1 = 9.26%). (Figure 1; Supplementary Tables S1–S8).

## 3. Results and Discussion

**Scheme 1:**
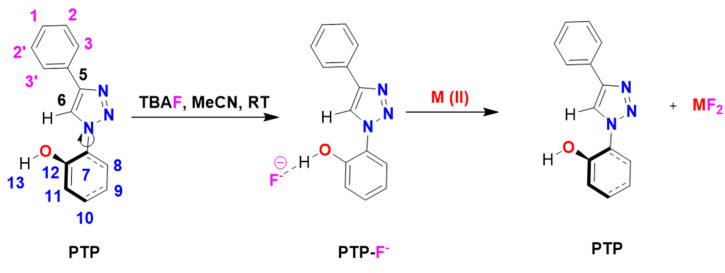
The proposed binding mode of PTP and fluoride anion and its reversibility after adding Zn (II) cation.

Our initial study on **PTP** targeting logic functions revealed that the “ON” process is monitored via a strong fluorometric response that is displayed when the sensor binds to fluoride (input 1) [15]. The TURN-OFF sequence, which is displayed by the quenching of the fluorescence, is triggered by the addition of metal cations such as zinc and copper in homogeneous media (input 2, Scheme 1). The color changes (under ambient light and UV lamp), UV-Vis, NMR, and single X-ray crystal studies aligned with our data. Logic operations on the PTP sensor were possible only because of its reversible abilities. 

### 3.1. Photophysical Studies

#### 3.1.1. A Combination of PTP, TBAF, and Zn^2+^

**Figure 2:**
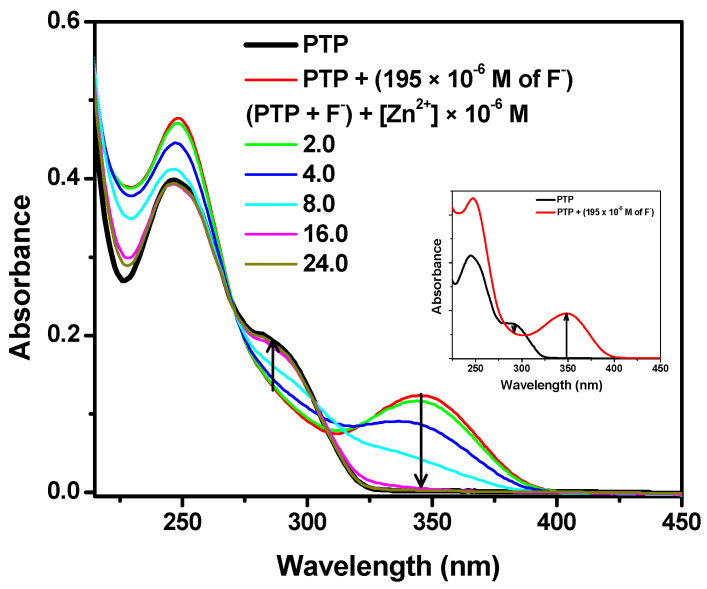
UV-Vis absorption spectral titrations of **PTP** (~1.7 × 10^−5^ mol/L) with the addition of Zn (II) perchlorate to **PTP**-TBAF ensemble in acetonitrile (TBAF was in 12 mole equivalents with respect to **PTP**). Concentrations of Zn (II) (0.12→1.4 mole equivalents of Zn^2+^ with respect to **PTP**) are provided in the legends. Inset shows the absorption spectra of **PTP** (black line) and **PTP** with fluoride (red line).

The optical switching phenomenon was studied on the fully characterized **PTP** molecule [15]. **PTP**’s lowest energy band in acetonitrile occurs ~290 nm at the π-π* transition of the substituted phenyl ring. The molecule showed a modulated spectrum with the appearance of a new absorption peak around 345 nm in the presence of F^−^, H_2_PO_4_^−^, and AcO^−^ anions (Supplementary Figure S3) [15]. Compared to the other contenders, the response with F^−^ was the most sensitive. Hence, the molecular switch experiments were conducted specifically with fluoride. Interestingly, when the probe was bound to F^−^, the addition of metal ions changed the spectral property at this stage. A gradual addition of Zinc perchlorate (Zn^2+^), in particular, led to the recovery of **PTP**’s original absorption (Figure 2). In a parallel control experiment, it was observed that **PTP**’s absorption was unmodified in the presence of Zn^2+^ alone. Hence, the spectral changes in the UV after the addition of F^−^ generated the INH logic gate (higher wavelength, λ_max_ = 345 nm); on the other hand, the reversibility after the addition of Zn^2+^ is complementary with the IMP logic gate (lower wavelength, λ_max_ = 290 nm).

Similar UV-Vis spectra were observed upon the respective addition of 5 equivalents of Ag^+^, Al^3+^, Cd^2+^, Co^2+^, Cr^3+^, Fe^3+^, Fe^2+^, Li^+^, Mg^2+^, and Ni^2+^ (Supplementary Figure S4a). Observations with Cu^2+^ are drastically different from the other metals due to an independent binding interaction of **PTP** with Cu (II) ion. This occurrence is described separately in Section 3.1.2.

As it can be observed that the optical outputs are solely controlled by the anion/cation inputs, a simultaneous study was made to explore the fluorescence response of **PTP** with the ions. The probe, **PTP**, is non-fluorescent by itself. However, the addition of F^−^ pronounces the fluorescence of the molecule, peaking around 430 nm in the same solvent. The emission of **PTP** in the presence of F^−^ was drastically quenched by adding metal ions Al^3+^, Cd^2+^, Co^2+^, Cr^3+^, Cu^2+^, Fe^3+^, Fe^2+^, Li^+^, Mg^2+^, Ni^2+^, and Zn^2+^ (Supplementary Figure S4b). The fluorescence resembles **PTP**’s original spectrum. Interference was observed from the Ag^+^, in which the molecule retained some of its fluorescence. However, with an elongated time, it followed complete quenching of fluorescence.

**Figure 3:**
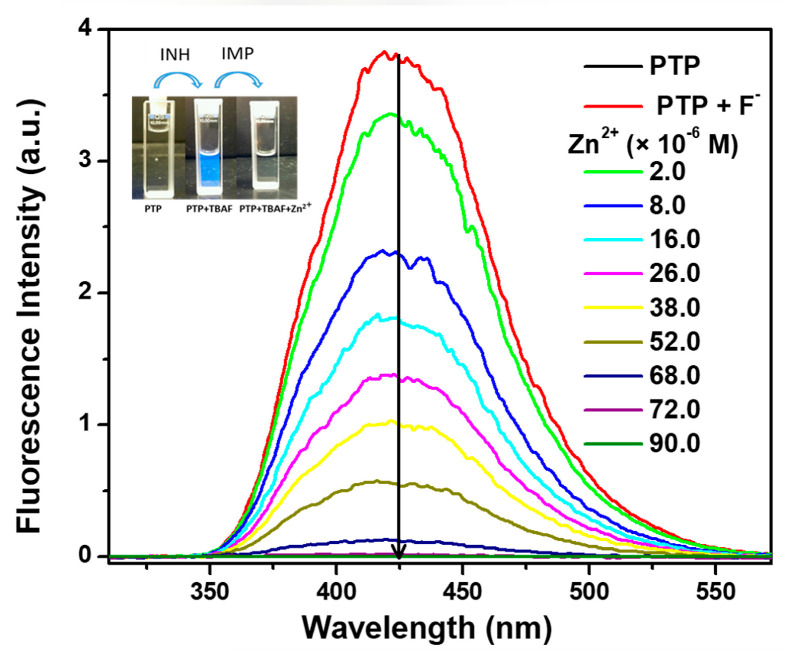
Fluorescence spectral variation of **PTP** (~1.7 × 10^−5^ mol/L) with the addition of Zn (II) perchlorate to **PTP**–TBAF mixture in acetonitrile. The concentrations of Zn^2+^ (0.12→5.3 mole equivalents of Zn^2+^ with respect to **PTP**) are provided in the legends, λ_exc_ = 290 nm. Image in the inset represents the fluorescence of **PTP**, **PTP**-F^−^, and **PTP**-F^−^-Zn^2+^ under the long-wavelength (365 nm) UV lamp.

The reversibility in the fluorescence response of the molecule was supported by conducting a fluorometric titration of **PTP**-F^−^ adduct with an increasing concentration of Zn^2+^. The results indicated a gradual decrease in the emission of **PTP**-F^−^ upon the incremental addition of Zn^2+^ ions (Figure 3). This validated that the original non-fluorescence property of **PTP** is recovered. The fluorosensing activity of **PTP** is also substantiated by the color change under the UV lamp (Figure 3 inset). As compared to **PTP**, a well-discriminated bright blue fluorescence of the **PTP**–F^−^ complex was displayed when the solution was observed under a long-wavelength UV lamp (~365 nm). Upon the addition of Zn (II) perchlorate, the fluorescence of the solution diminished, resembling the original color of **PTP** (Figure 3). The equilibrium constant for this two-step process was calculated following the methods described by Ramette et al. [30]. The equilibrium constant (K_eq_ = 1.67 × 10^−5^) was evaluated using the fluorescence of **PTP** at 430 nm when Zn^2+^ was gradually added to the **PTP**-fluoride adduct (Supplementary Figure S11a,b) [31].

#### 3.1.2. A Combination of PTP, TBAF, and Cu^2+^

**Figure 4:**
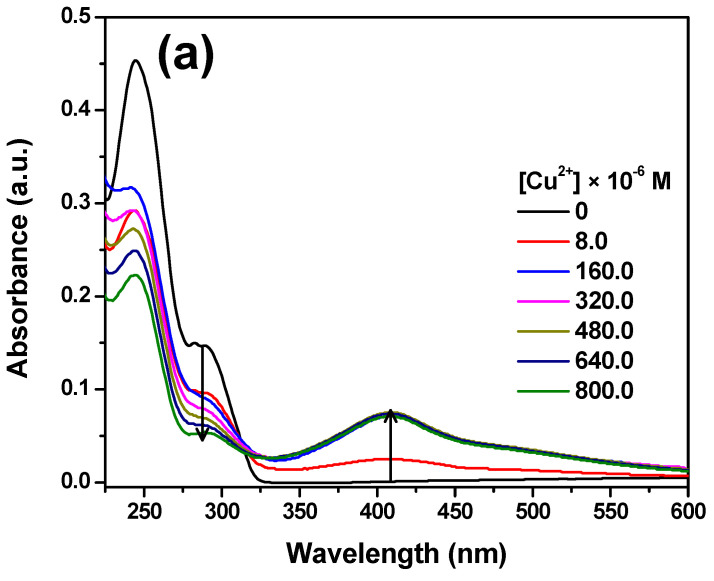

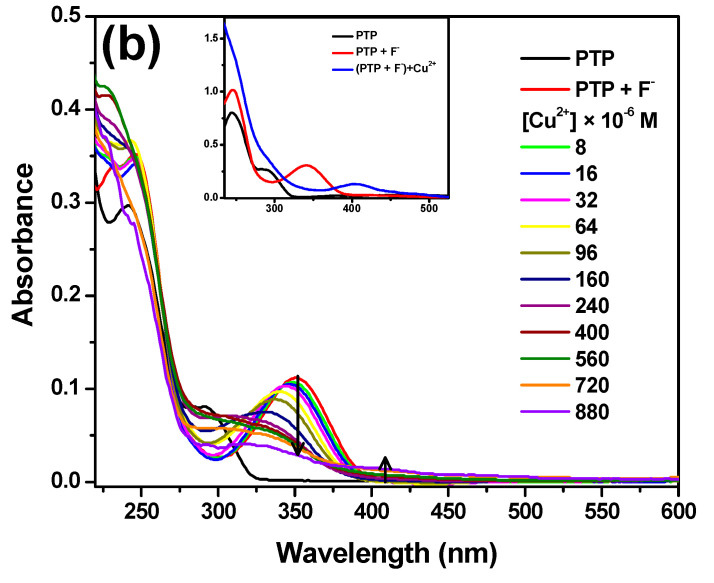
Absorption spectral variation of: (**a**) **PTP** (~1.7 × 10^−5^ mol/L) with the addition of copper (II) perchlorate hexahydrate (0→47 mole equivalents of Cu^2+^ with respect to **PTP**) in acetonitrile; and (**b**) **PTP** + TBAF + Cu^2+^ (0.48→52 mole equivalents of Cu^2+^ with respect to **PTP**). Copper salt concentrations are provided in the legends.

The UV-Vis spectra of the chemosensor **PTP** showed a gradual decrease in its signature low-energy peak at 290 nm when Cu (II)- perchlorate was added. However, the simultaneous growth of another band around 408 nm going through an isosbestic point at 323 nm was observed. This indicated the formation of a new species (Figure 4a), correlating the previous observations with the bis-appended-1,2,3-triazole and phenanthrene derivatives of **PTP** in the presence of Cu^2+^ [16,19]. In the phenanthrene derivative, the triazole exhibited 1:1 binding interaction [19] due to the charge transfer between triazolyl nitrogen and the metal ion forming a new absorption at 415 nm. 

**Figure 5:**
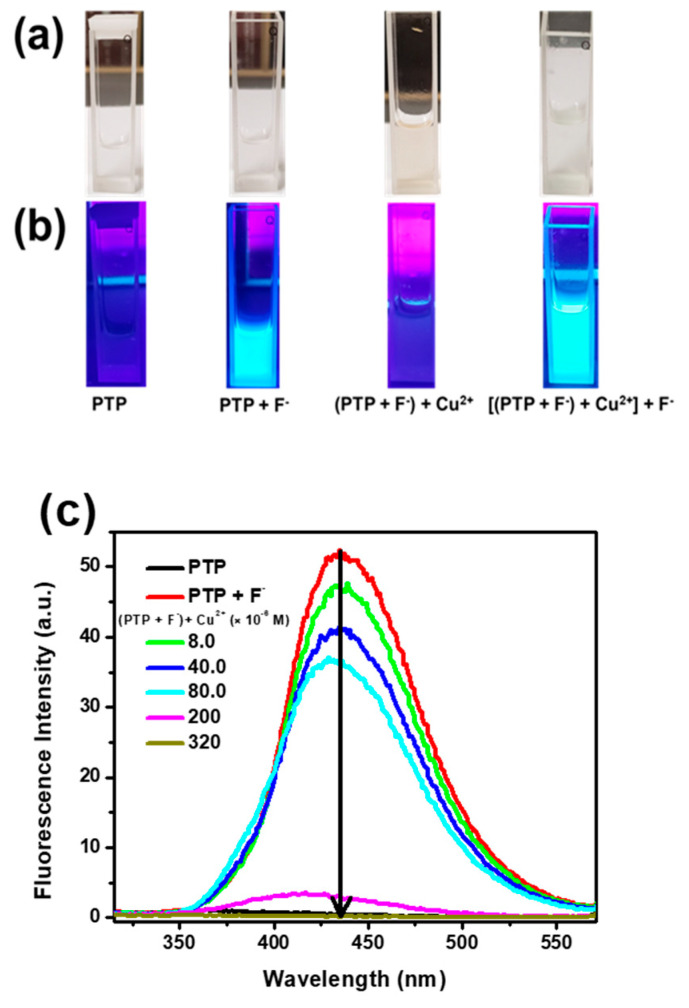
OFF-ON-OFF cycle of **PTP** with TBAF and Cu (II) perchlorate under (**a**) ambient light and (**b**) UV lamp. (**c**) Fluorescence spectra of **PTP** (~5.0 × 10^−5^ mol/L) with the addition of Cu (II) perchlorate (0.48→1.9 mole equivalents of Cu^2+^ with respect to **PTP**) to **PTP**–TBAF mixture in acetonitrile. The concentrations of Cu^2+^ are provided in the legends, λ_exc_ = 290 nm.

Similar to the **PTP**-F^−^-Zn^2+^ study, the reversibility of the process was manifested with the **PTP**-F^−^-Cu^2+^ combination. After **PTP** was saturated with the titration of F^−^, the addition of Cu (II) perchlorate to the above solution resulted in the disappearance of the absorption at 345 nm. The gradual occurrence of the 408 nm band is attributed to **PTP**’s binding with Cu^2+^ (Figure 4b). This accounted for competitive binding to **PTP** among F^−^ and Cu^2+^. A unique pathway was followed for this combination. Under ambient light, **PTP** and **PTP** + F^−^ were colorless. Adding copper (II) perchlorate generated a light brown coloration to the solution (Figure 5a). This observation corresponded to the earlier reports on the phenanthrene-based triazole sensor, thereby confirming the binding interaction of Cu^2+^ with the triazole nitrogen [19]. The addition of TBAF to this solution, however, resulted in a discoloration resembling that of the **PTP** solution. The equilibrium constant (K_eq_ = 1.52 × 10^−4^) for this process was assessed by taking a similar approach as the **PTP**–fluoride–Zn system and measuring **PTP’s** absorbance at 345 nm (Supplementary Figure S11c,d). In fluorescence, under UV-lamp, a similar OFF-ON-OFF cycle with Cu (II) perchlorate as that of Zn (II) perchlorate is depicted in Figure 5b. The fluorescence spectra (Figure 5c) of **PTP** supported the observation.

### 3.2. NMR Studies

#### 3.2.1. A Combination of PTP, TBAF, and Zn^2+^

**Figure 6:**
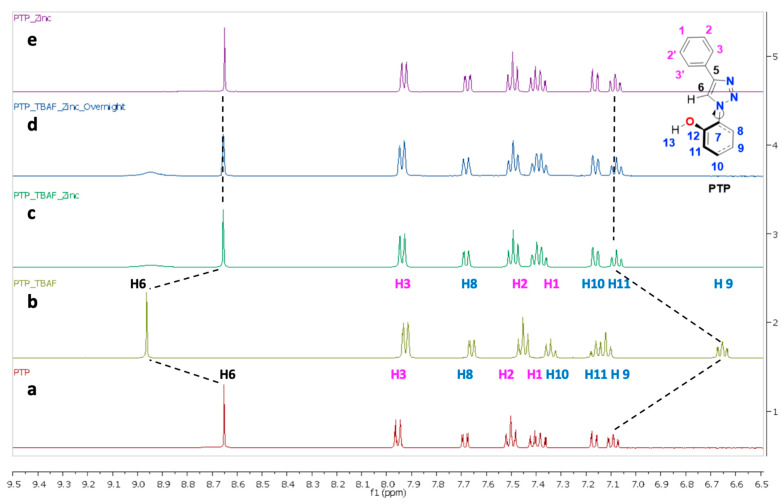
Stacked ^1^H-NMR (partial, 400 MHz, 1.8 × 10^−1^ mol/L in CD_3_CN, RT, from bottom to top) spectra for pure **PTP** ((**a**), bottom), **PTP** with TBAF ((**b**), 1 eq.), and PTP with TBAF and Zinc perchlorate ((**c**), immediate) (1 eq.), **PTP** with TBAF and Zinc perchlorate ((**d**), overnight) (1 eq.), and **PTP** and Zinc perchlorate only ((**e**), top).

The ^1^H-NMR investigations showed that the system is “resettable” (Figure 6) and confirmed the results obtained from UV and fluorescence studies. With F^−^ anion (input 1), the phenolic and triazole protons were significantly shifted up- and downfield for the **H9** and **H6** protons, respectively. The subsequent addition of Zn^2+^ (input 2) showed that the probe can be recovered “unharmed” in its original form. The metal ion’s effect was solely due to the scavenging of fluoride by Zn(II) forming ZnF_2_. There is no binding interaction with Zn^2+^.

The ^1^H NMR studies provided further support to understand the logic functions. The triazole proton (**H6**) at *δ* 8.65 ppm (Figure 6a) shifted to *δ* 8.95 ppm (Figure 6b), complementing the INH logic gate. The **H6** proton switched back after the addition of zinc metal ion following the IMP gate (Figure 6c,d). In the presence of only zinc ion, the probe, **PTP**, remained unchanged (Figure 6e). This corroborates the observed UV-Vis absorption and fluorescence changes in the earlier section. 

**Figure 7:**
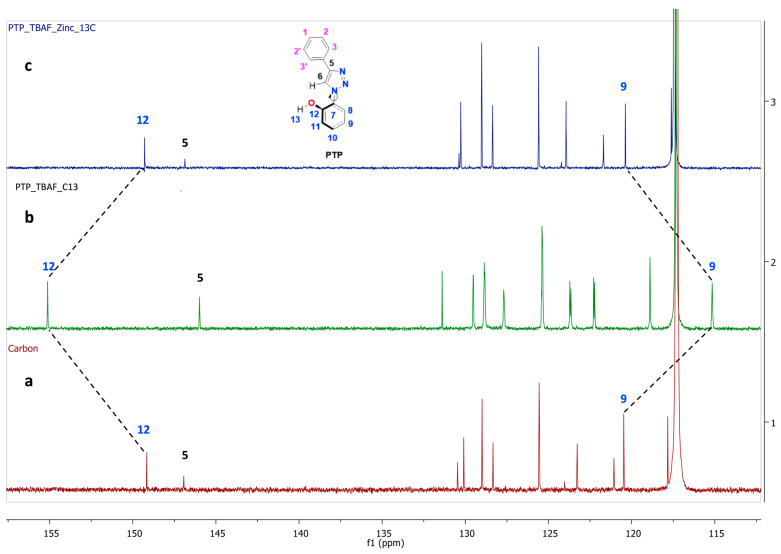
Stacked ^13^C-NMR (partial, 400 MHz, 1.8 × 10^−1^ mol/L in CD_3_CN, RT, from bottom to top) spectra for pure **PTP** ((**a**), bottom), **PTP** with TBAF ((**b**), 1 eq.), and **PTP** with TBAF and Zinc perchlorate ((**c**), 1 eq., top).

The ^13^C NMR results (Figure 7) are in complete agreement with the results from the ^1^H NMR. For this study, the **C12** and **C9** were targeted due to the significant downfield and upfield movement shown by these signals, respectively. The carbon signal for C12 showed a peak at *δ* 149.1 ppm (Figure 7a), which induces a downfield shift to *δ* 156.2 ppm after the addition of TBAF (Figure 7b). This fulfils the INH gate function. The peak is restored to the original position *δ* 149.1 ppm after the addition of Zn (II) perchlorate (Figure 7c), supporting the IMP logic gate. A similar upfield pattern was observed in the C9 signal from *δ* 120.5 ppm to *δ* 115.2 ppm. The shift is approximately 5–7 ppm on either side.

#### 3.2.2. A Combination of PTP, TBAF, and Cu^2+^

**Figure 8:**
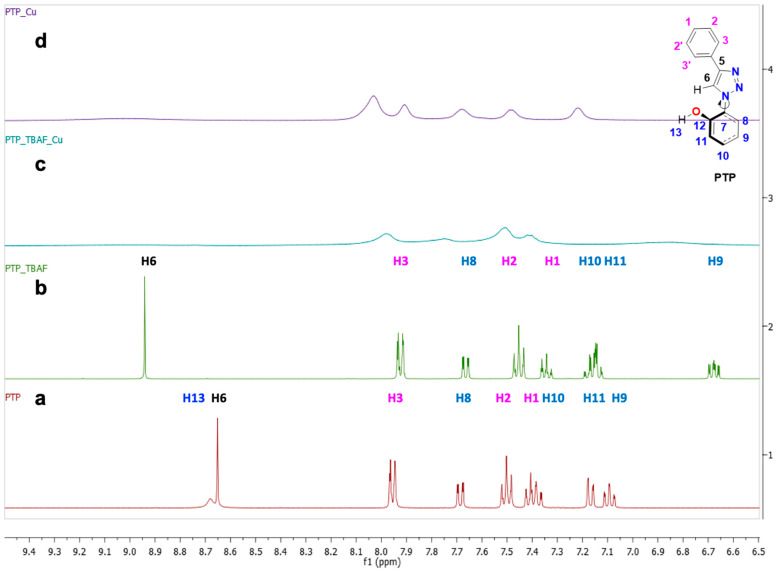
Stacked ^1^H-NMR (partial, 400 MHz, 1.8 × 10^−1^ mol/L in CD_3_CN, RT, from bottom to top) spectra for pure **PTP** ((**a**), bottom), **PTP** with TBAF ((**b**), 1 eq.), **PTP** with TBAF and Copper perchlorate ((**c**), 1 eq.), and **PTP** and Copper (II) perchlorate only ((**d**), top, 1 eq.).

The ^1^H-NMR study with the **PTP**–TBAF complex after adding copper perchlorate showed a signal broadening of the peaks (Figure 8) due to the paramagnetic effect. The copper salt overwhelmed the spectrum with additional equivalents of copper (II) [16]. A solution of **PTP** and Cu^2+^ also showed the initiation of broadening. The ensemble of all three reactants made it difficult to characterize through NMR spectroscopy Figure 8. The other findings outlined in this paper from fluorometric and color studies support the logic gate function for copper (II) ions as well. 

#### 3.2.3. A Combination of PTP, TBAF, and Other Metal Salts

**Figure 9:**
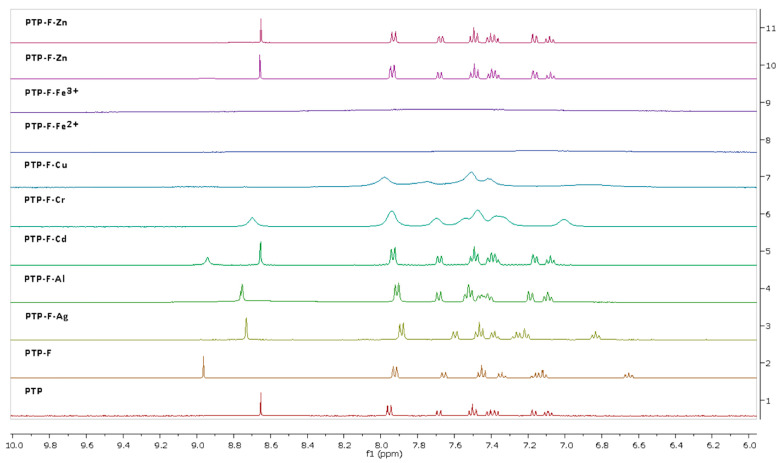
Stacked ^1^H-NMR (partial, 400 MHz, 1.8 × 10^−1^ mol/L in CD_3_CN, RT, from bottom to top) spectra for pure **PTP** (bottom), **PTP** with TBAF (1 eq.); and **PTP** with TBAF and upon addition of 1.0 equivalent amounts of selected metal perchlorates (Ag^+^, Al^3+^, Cd^2+^, Cr^2+^, Cu^2+^, Fe^2+^, Fe^3+^, Zn^2+^ (immediate), Zn^2+^ (top, overnight)).

In order to obtain further information on the interaction of the **PTP**–fluoride complex, ^1^H-NMR experiments were conducted with different metal ions to design complementary circuits. Other than the zinc and copper ions discussed above, biologically and environmentally relevant metal ions such as Ag^+^, Al^3+^, Cd^2+^, Cr^3+^, Fe^2+^, Fe^3+^ etc. were tested (Figure 9). The **PTP** molecule in CD_3_CN was mixed with TBAF (1 eq.) and immediately treated with various equivalents of the metal salts. 

Metal ions Ag^+^, Al^3+^, and Cd^2+^ showed the reversible pattern for the triazole proton (**H-6**), the -OH (**H-13**), and other phenolic protons. Simultaneously, the triazole C_sp2_-H (**H-6**) proton was deshielded from *δ* 8.65 ppm to *δ* 8.95 ppm. After adding Ag^+^ salt to the **PTP**–TBAF complex, the **PTP** molecule did not completely reset immediately like the zinc salt. Our hypothesis is that with an ample amount of time, the system will be restored eventually (Supplementary Figure S5). The results with Ag^+^ compliment the studies with UV absorption (Supplementary Figure S4b). The addition of the aluminum (III) cation was able to bring the **PTP** to its original form (immediate), confirming an IMP/INH logic behavior. If the same complex (**PTP**-TBAF-Al^3+^) was tested for overnight reading, it resulted in the broadening of peaks (Supplementary Figure S6). This phenomenon was not seen in Zinc perchlorate (Figure 6d). Cadmium (II) did reset the PTP molecule but with an additional unknown broad signal at 8.95 ppm (Supplementary Figure S7).

Metal ions such as chromium (III) and copper (II) showed a severe broadening of the signals (Supplementary Figure S8, Figure 8). Ions iron (II) and iron (III), however, did not show signals due to the paramagnetic impact (Supplementary Figures S9 and S10). These ions could not provide much information on the interaction with the **PTP**–fluoride conjugate. Given all the information in hand obtained from NMR studies, zinc perchlorate metal cation was the metal of choice (top two spectrums in Figure 9 (immediate and overnight). In the future, detailed studies with these additional metal salts may result in a variety of logic gate or molecular switch applications (Figure 9).

### 3.3. Molecular Logic Gate Property of PTP with Fluoride and Metals

The probe **PTP** displayed a molecular system with specific colorimetric, fluorometric, NMR signals, and color studies with fluoride and metal ions. A complex logic operation was obtained in the presence of multi-inputs (F^−^ or Zn^2+^ or Cu^2+^). This led to the construction of IMPLICATION (IMP), INHIBIT (INH), and OR gates.

**Figure 10:**
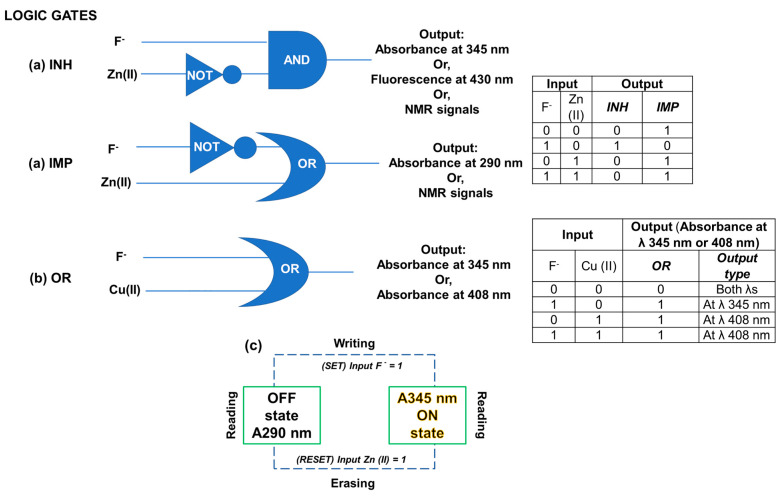
For the **PTP**–fluoride–zinc system, (**a**) the INH gate was constructed with **PTP**’s absorption at 345 nm, emission at 430 nm, and NMR signals (*δ* 8.95 ppm for ^1^H and *δ* 156.2 ppm for ^13^C). IMP gate was developed using the absorption output of **PTP** at 290 nm and the NMR signals (*δ* 8.65 ppm for ^1^H and *δ* 149.1 ppm for ^13^C). For the **PTP**–fluoride–copper system, (**b**) the OR gate represents the absorption outputs of **PTP** at 345 nm and 408 nm. Corresponding truth tables are depicted beside each function. (**c**) Feedback loop with sequential and reversible logic operations presenting “Write-Read-Erase-Read” behavior.

For the **PTP**–F^−^–Zn^2+^ system, the IMP gate was fabricated with the colorimetric output of **PTP** (probe only) and with the absorption at λ_290nm_ and the NMR signals at *δ* 8.65 ppm (^1^H) and *δ* 149.1 ppm (^13^C) in the absence and presence of inputs F^−^ or Zn^2+^. On the other hand, the INH gate focused on all the analytic outputs. **PTP**’s response at λ_345nm_ in absorption, λ_430nm_ in emission spectra, and NMR signals at *δ* 8.95 ppm (^1^H) and *δ* 156.2 ppm (^13^C) were recorded for this gate. The absorption of **PTP** at 290 nm can be read in the absence of any inputs (e.g., F^−^ or Zn^2+^). This corresponded to the output “1” in the IMP gate (Figure 10a). Since **PTP** is non-fluorescent, at this input condition, the fluorescence is “OFF” when the molecule is excited at 290 nm. This showed “0” as the output in INH (Figure 10a). However, in the presence of input F^−^, the absorbance at 290 nm decreased (with a concomitant emergence of a new band at 345 nm), correlating output “0” in the IMP gate. However, in INH, it is “1” due to the turned “ON” fluorescence of **PTP** at 430 nm complementing the appearance of the 345 nm band in the absorption spectrum at this condition. When the probe (**PTP**) is subjected to another sole input Zn^2+^, the absorption (λ_290nm_) and emission properties of the original **PTP** are retained. These resemble outputs “1” and “0” in IMP and INH gates, respectively. Finally, in the presence of both the inputs (F^−^ and Zn^2+^), **PTP**’s absorption at 290 nm is regained with a parallel observation of TURN-OFF fluorescence at λ_430nm_. Below is the truth table shown for the **PTP** sensor; the chemical inputs are F^−^ and Zn (II), and the output signals are in the form of absorption and fluorescence change (under a UV lamp). We can then encompass these output circuits to an electronic equivalent shown below in Figure 10. 

With **PTP**-F^−^-Cu^2+^, the outputs followed a basic OR gate based on the modulation in the absorption spectrum of **PTP** (Figure 10b) [32]. The absorbance due to fluoride binding at 345 nm and that of Cu^2+^ at 408 nm were recorded to construct the gate. In the absence of any inputs (F^−^ or Cu^2+^), the output signal (at 345 nm or 408 nm) was “0”. It was “1” in the presence of F^−^ or Cu^2+^ individually. When Cu^2+^ was added to the **PTP**-fluoride ensemble, the 408 nm absorption was still retained, showing an output “1”. The truth table for this sensing pattern is represented in Figure 10b. In fluorescence, the **PTP**-F^−^-Cu^2+^ followed the same INH gate as **PTP**-F^−^-Zn^2+^ with the “OFF-ON-OFF sequence”. It is quite interesting that **PTP** exhibited single-molecule differential sensing with two inputs utilized for constructing multimodal gates [33].

Thus, this assembly of the “OFF-ON-OFF” fluorescence simulates the “write-read-erase-read” function that can generate a reversible and reconfigurable sequence in a feedback loop (Figure 10c) [34]. Considering the fluorescence output of **PTP** at 430 nm, when F^−^ was used as a set input, the fluorescence was enhanced, and this information was stored as “written”. The encoded information was “erased” by adding Zn^2+^ as “reset input” through fluorescence quenching at 430 nm [24,35]. The versatility of this molecular system was checked by repeating the write/erase loop that conclusively demonstrated the occurrence of the “OFF-ON-OFF” fluorescence intensity. The combinatorial logic gates (IMP/INH) and the “multi-write” ability of this probe with different inputs paved the road toward data storage.

## 4. Conclusions

In summary, 1,2,3 triazole derivative (**PTP**) synthesized through “click chemistry” is capable of detecting F^−^ and Zn^2+^/Cu^2+^ ions. The **PTP**’s selectivity towards fluoride and its reversibility with metal ions has been studied. With fluoride, **PTP** exhibited a strong TURN ON fluorometric change; with zinc (II)/copper (II), **PTP** exhibited a TURN-OFF fluorescence. **PTP** established a strong reversible system based on two inputs complementary to the IMP/INH logic functions for F^−^/Zn (II). A basic OR gate is manifested by the ON and OFF sequence with Cu (II). The fluorometric molecular switching by a well-planned mix of anions and cations in the triazoles probe can lead to applications such as molecular keypad locks, security systems, memory devices, and molecular machines, and target the Write-Read-Erase-Read memory functions. Furthermore, our simple and effective sensors can be useful in optical and electronic systems, which can be tweaked at the molecular level to modify their reversible switching behavior to unambiguously select for the Zn^2+^/Cu^2+^ or fluoride anion under investigation. 

## Data Availability

Not applicable.

## Supplementary Materials

The following supporting information can be downloaded at: https://www.mdpi.com/article/10.3390/s23157000/s1, Included supporting information for details on related content. For the single crystal X-ray data in the Cambridge Crystallographic Data Centre (CCDC) the deposition # is 2282916. References [15,36,37,38,39] are cited in the supplementary materials.

## Abbreviations

PTP	1,4-diaryl-1,2,3-triazole with a phenol moiety
HRMS	High-resolution mass spectrometry
MP	Melting point
NMR	Nuclear Magnetic Resonance
TMS	Tetramethyl Silane
INH	Inhibit
IMP	Implication
TBA	tetrabutylammonium
DMSO	dimethylsulfoxide
CD_3_CN	deuterated acetonitrile
UV-Vis	Ultraviolet visible
