# Supplementary material for: 1,2,3-Triazoles: Controlled Switches in Logic Gate Applications
Source: Sensors (Basel). 2023 Aug 7;23(15):7000. doi: 10.3390/s23157000 (PMC10422406; doi:10.3390/s23157000)
Supplement: Supplementary file 1 [file sensors-23-07000-s001.zip › sensors-2509510-supplementary.pdf]

1,2,3-Triazoles: Controlled Switches in Logic Gate Applications

Debanjana Ghosh [1,2], Austin Atkinson [1], Jaclyn Gibson [1], Harini Subbaiahgari [2], Weihua Ming [1], Clifford Padgett [3], Karelle S. Aiken [1] and Shainaz M. Landge [1,*]

1 Department of Chemistry and Biochemistry, Georgia Southern University (Statesboro Campus), 521 College of Education Drive, Statesboro, GA 30460-8064, USA; dghosh@siue.edu (D.G.)
2 Department of Chemistry, Science Building West, Southern Illinois University Edwardsville, Edwardsville, IL 62026-1652, USA
3 Department of Chemistry and Biochemistry, Georgia Southern University (Armstrong Campus), 11935 Abercorn Street, Savannah, GA 31419, USA
* Correspondence: slandge@georgiasouthern.edu

Supporting Information

Table of Contents

Synthetic Scheme 2

^{1}H NMR spectrum of **PTP** 3-4

^{13}C NMR spectrum of **PTP** 5-6

Absorption spectra of **PTP**+F^-, $H_2PO_4^-$ and OAc^- 7

Absorption and Fluorescence spectra of **PTP**+F^- with metals 8

Stacked ^{1}H NMR spectrum of **PTP**+TBAF+Silver 9

Stacked ^{1}H NMR spectrum of **PTP**+TBAF+Aluminum 10

Stacked ^{1}H NMR spectrum of **PTP**+TBAF+Cadmium 11

Stacked ^{1}H NMR spectrum of **PTP**+TBAF+Chromium 12

Stacked ^{1}H NMR spectrum of **PTP**+TBAF+Iron(III) 13

Stacked ^{1}H NMR spectrum of **PTP**+TBAF+Iron(II) 14

Benesi-Hildebrand (B-H) plots and calculation of equilibrium constants for the PTP-Fluoride- metals combination 15-16

Single Crystal X-ray data 17-21

Synthetic Scheme:

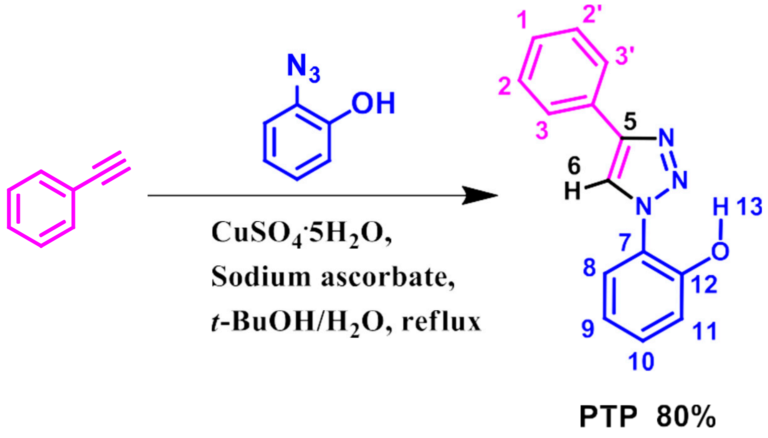

Scheme S1: The synthesis of **PTP** and its detailed characterization was published elsewhere by our group.[1]

Characterization of probe PTP:

^{1}H-NMR [400 mHz, CD_3CN] δ 8.68 (*s*, <u>H</u>O), 8.65 (*s*, 1-H), 7.96 (*dd*, 2-H, 1.3, 7.5Hz), 7.68 (*dd*, 1-H, 1.6, 8.0Hz), 7.50 (*t*, 2-H, 7.4Hz), 7.41 (*t*, 1-H, 7.5Hz), 7.39 (*dd*, 1-H, 1.6, 7.4Hz), 7.17 (*dd*, 1-H, 1.3, 8.3Hz), 7.09 (*td*, 1-H, 1.3, 7.4Hz); **^{13}C-NMR** [100 mHz, CD_3CN] δ 149.1, 146.9, 130.4, 130.1, 128.9, 128.3, 125.6, 124.0, 123.3, 121.1, 120.5, 117.8; **ESI-MS** for **PTP**: *m/z* = 238.05 $[M+H]^+$; *calculated* value for $C_{14}H_{11}N_3O$ = 237, *found* from experiment 238.05. **HRMS** $(M + H)^+$ *calculated* for $C_{14}H_{11}N_3O$ = 238.0975, *found* = 238.0965.

Reference:
1. Ghosh, D., Rhodes, S., Hawkins, K., Winder, D., Atkinson, A., Ming, W., Padgett, C., Orvis, J., Aiken, K. and Landge, S., **2015**. A simple and effective 1, 2, 3-triazole based "turn-on" fluorescence sensor for the detection of anions. *New Journal of Chemistry*, *39*(1), 295-303.

Nuclear Magnetic Resonance Spectroscopy Characterization of PTP:

PTP, ^{1}H NMR Spectrum

(a)

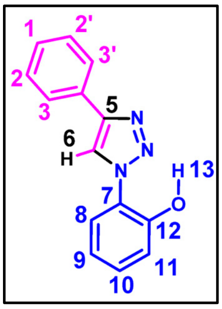

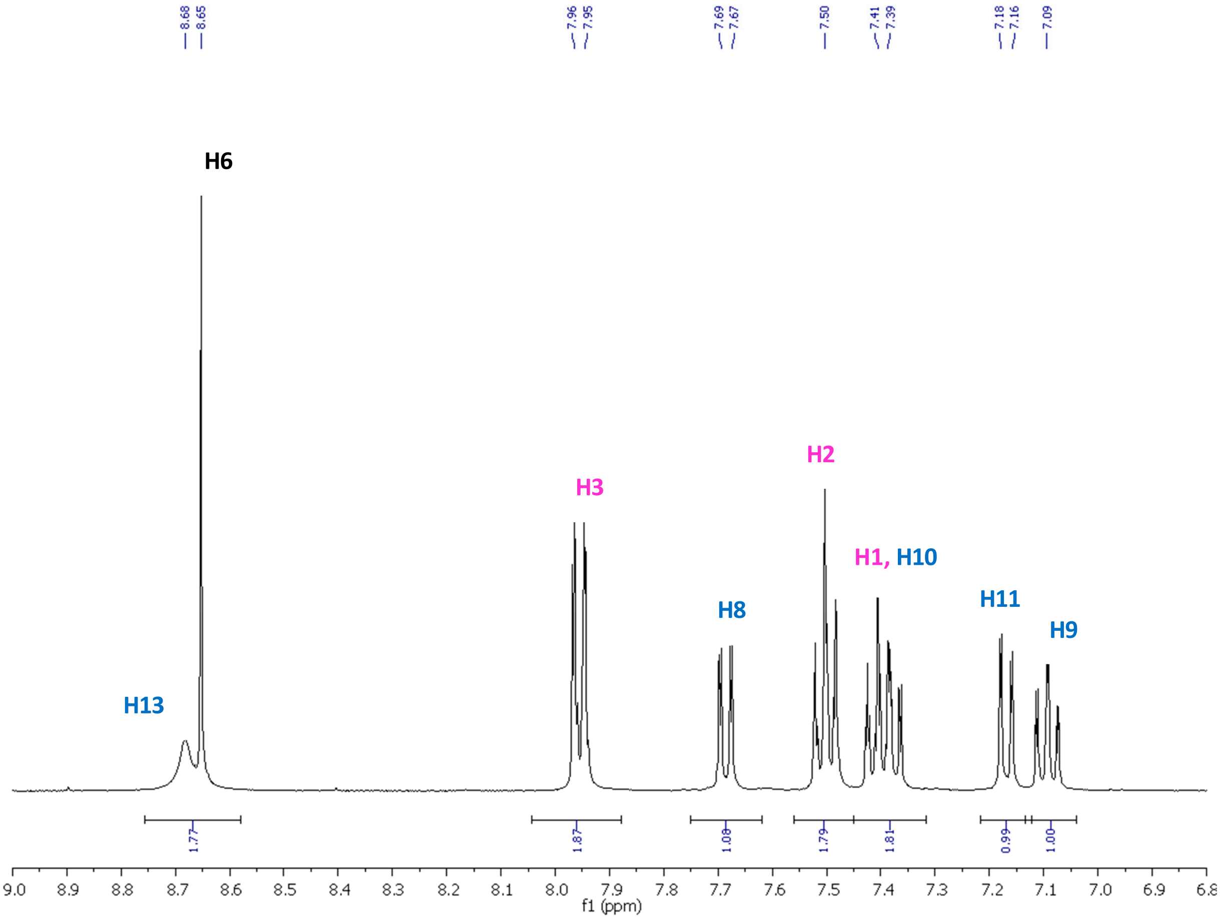

(b)

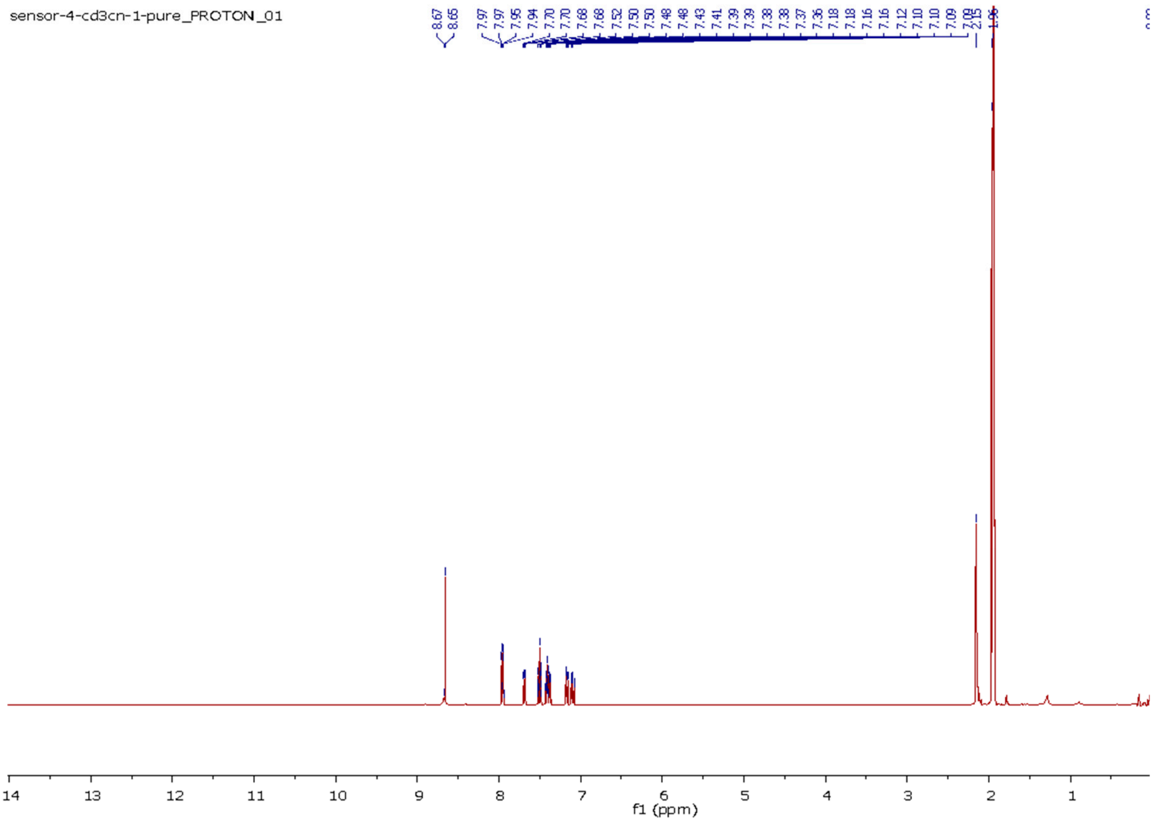

Figure S1: NMR spectra (^{1}H = 400 MHz, CD_3CN, RT) of **PTP**. (a) ^{1}H-NMR spectrum showing the expansion of aromatic region from 6.80 to 9.00 *ppm*. (b) Full ^{1}H-NMR spectrum of **PTP**.

PTP, ^{13}C NMR Spectrum

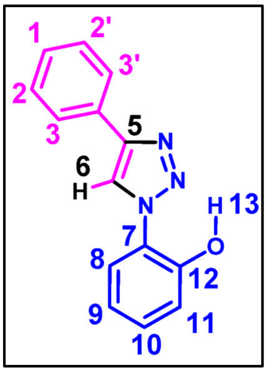

(a)

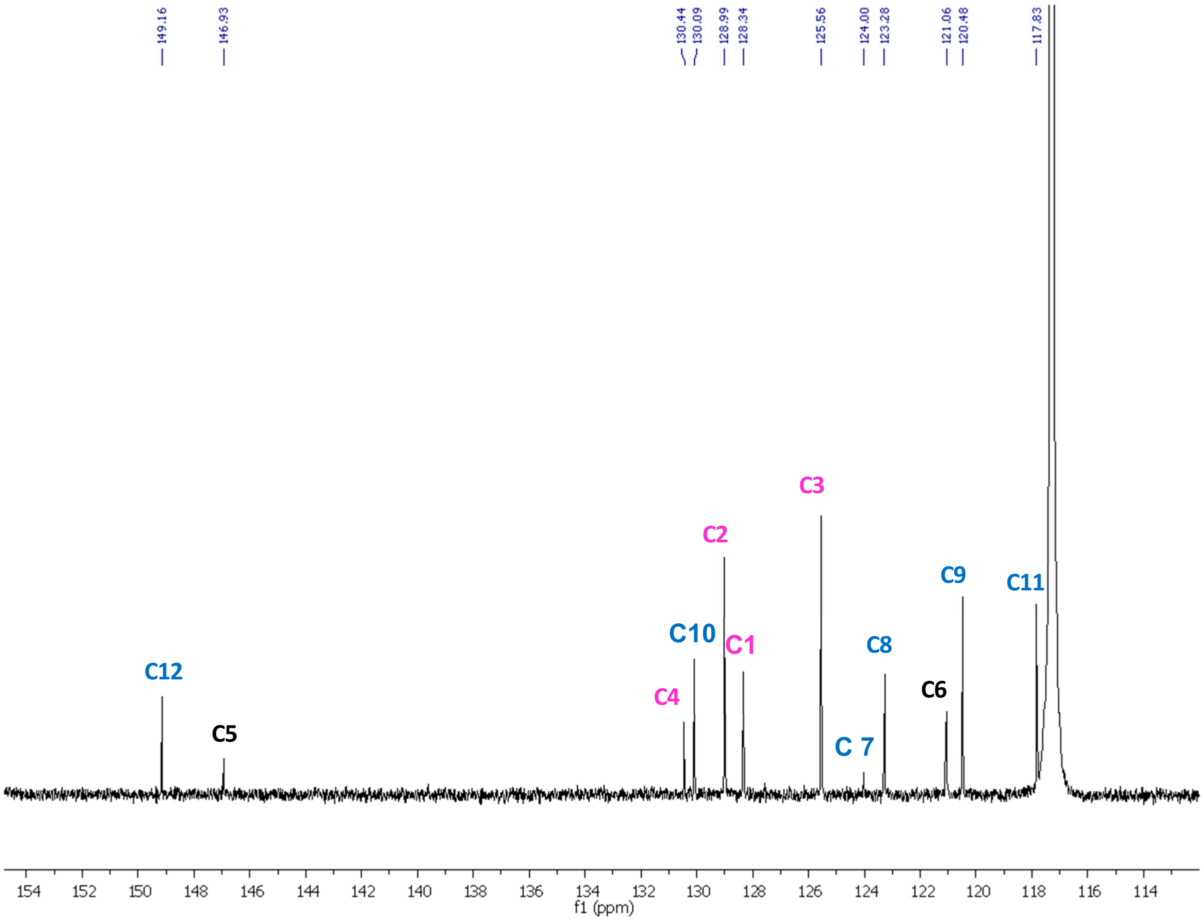

(b)

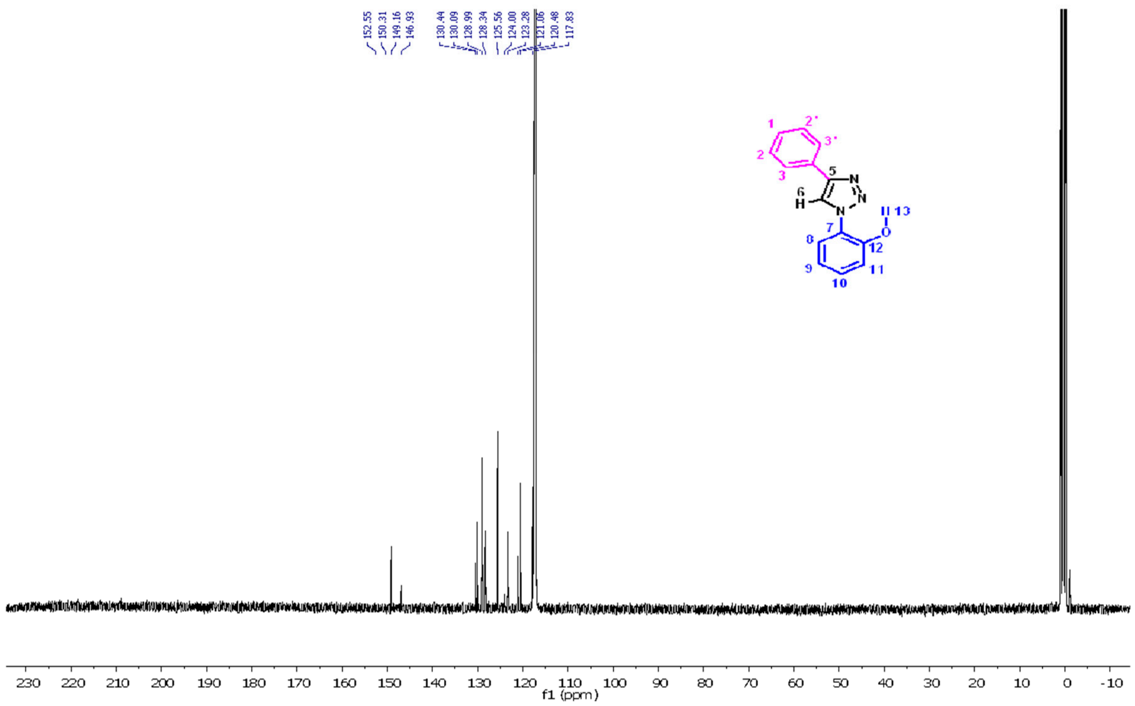

Figure S2: NMR spectra (^{13}C = 100 MHz, CD_3CN, RT) of **PTP**. (a) ^{13}C-NMR spectrum showing the expansion from 112-155 *ppm*. (b) Full ^{13}C-NMR spectrum of **PTP**.

Absorption spectra of PTP+F^- , $H_2PO_4^-$, and OAc^-

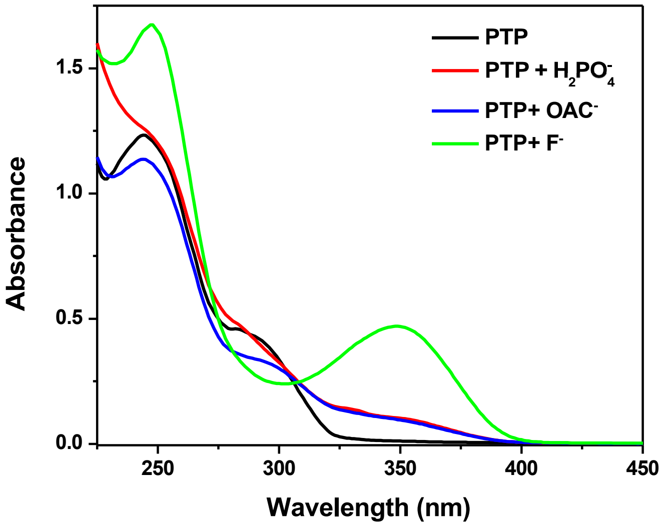

Figure S3: Absorption spectra of **PTP** (~1.7 × 10^{-5} mol/L) (black line) with the addition of the tetrabutylammonium (TBA) salts of fluoride (green line), dihydrogen phosphate (red line), and acetate (blue line) in acetonitrile.

Absorption and Fluorescence spectra of PTP+F^- with metals

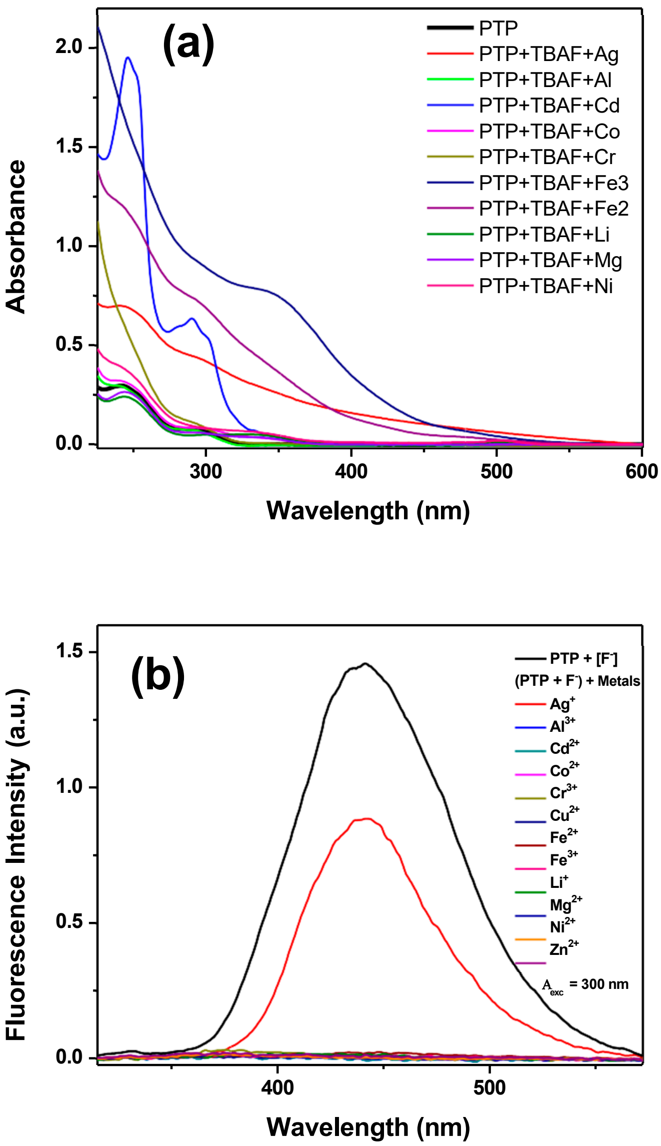

Figure S4: (a) Absorption and (b) fluorescence spectral variation of **PTP** when 2.0×10^{-5} mol/L of metal perchlorate salts were added to the **PTP**-fluoride ensemble in acetonitrile.

PTP+TBAF+Silver

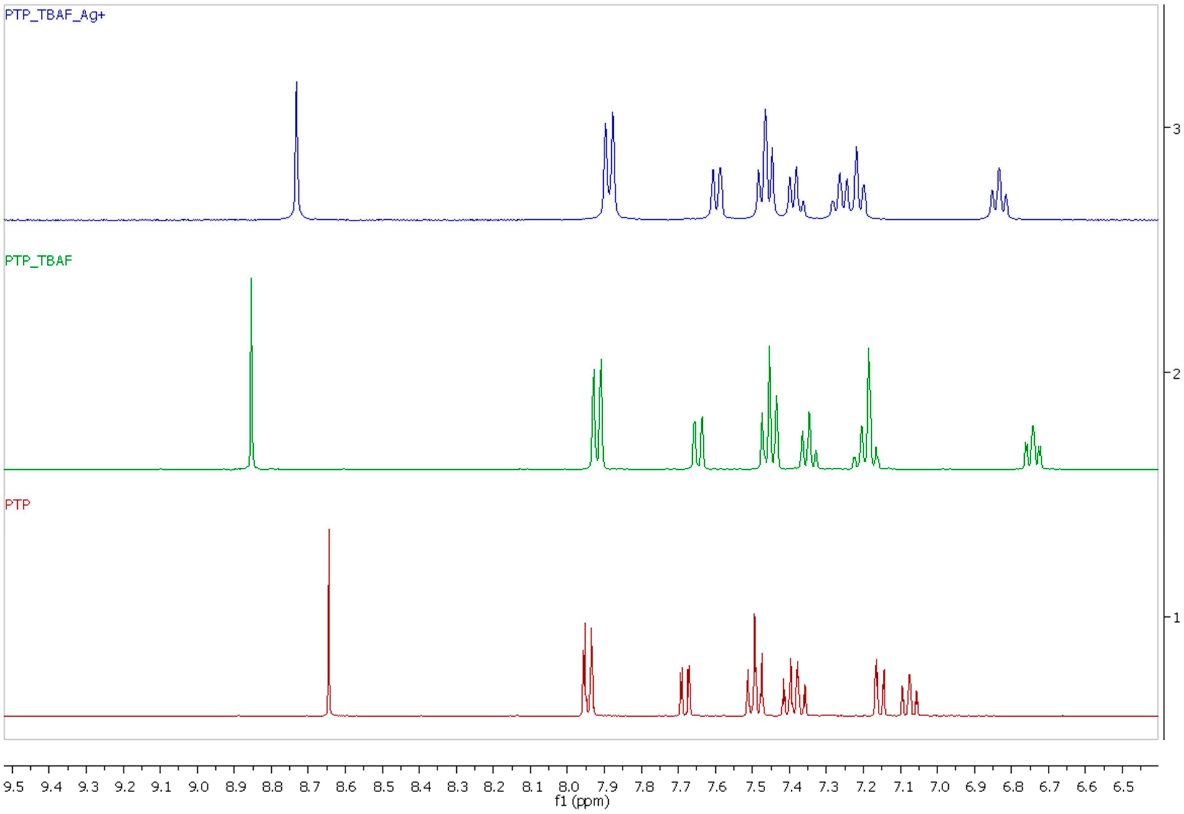

Figure S5: Stacked ^{1}H-NMR spectra (^{1}H = 400 MHz, CD_3CN, RT) of **PTP, PTP+TBAF,** and **PTP+TBAF+Ag$^+$** showing the expansion of aromatic region from 6.40 to 9.50 *ppm*.

PTP+TBAF+Aluminum

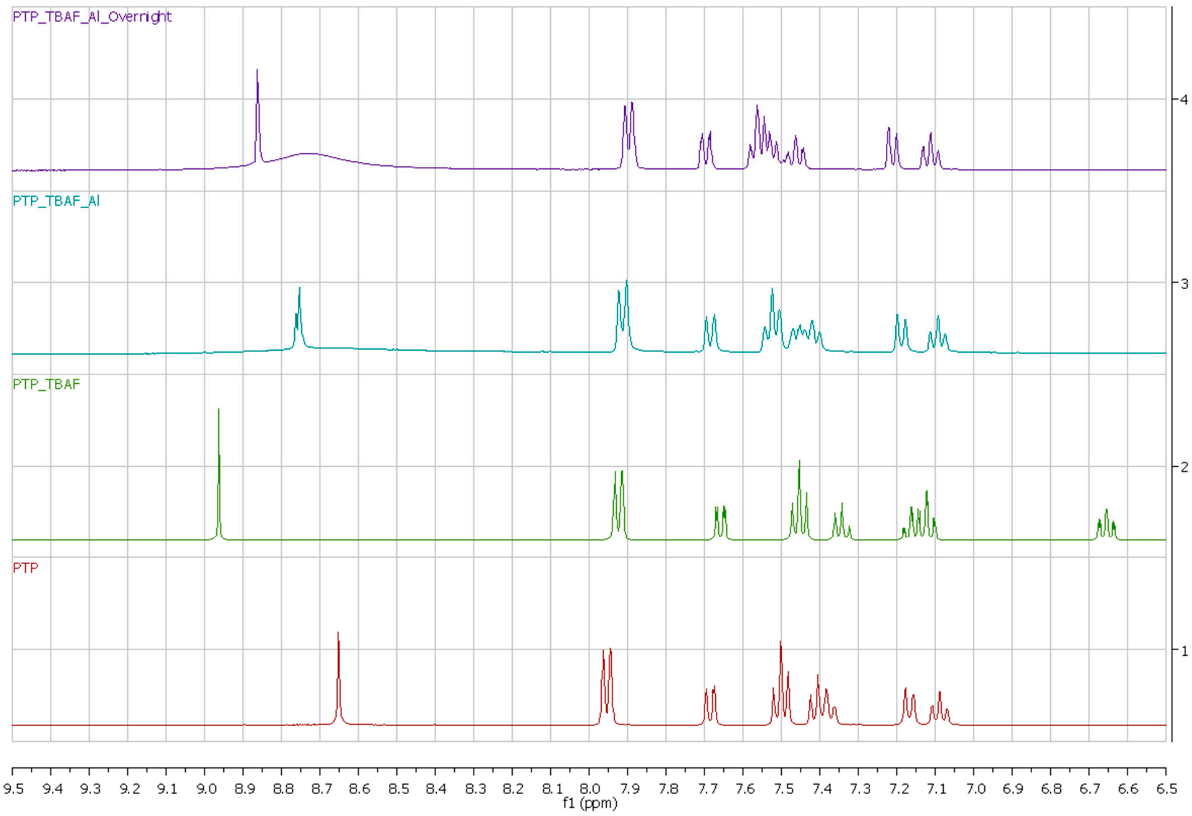

Figure S6: Stacked ^{1}H-NMR spectra (^{1}H = 400 MHz, CD_3CN, RT) of **PTP, PTP+TBAF, PTP+TBAF+Al^{3+} (*immediate*),** and **PTP+TBAF+Al^{3+} (*overnight*)** showing the expansion of aromatic region from 6.50 to 9.50 *ppm*.

PTP+TBAF+Cadmium

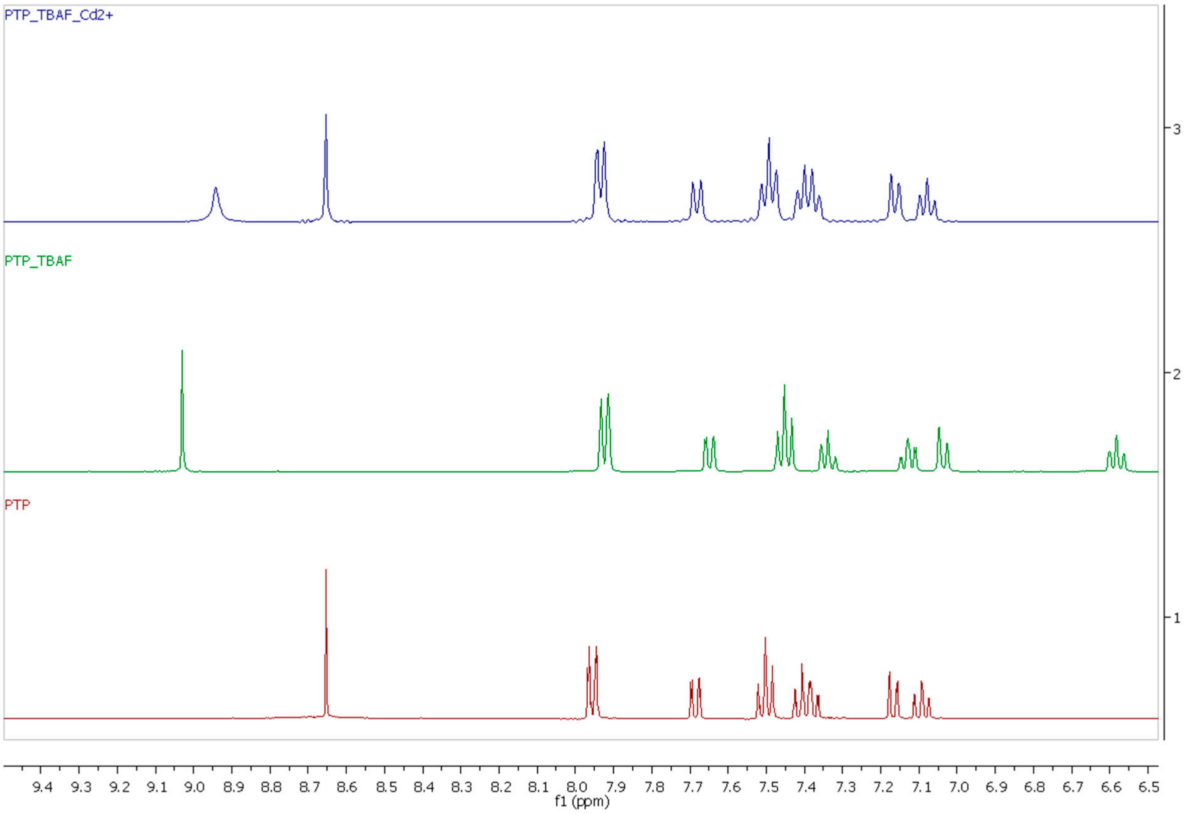

Figure S7: Stacked ^{1}H-NMR spectra (^{1}H = 400 MHz, CD_3CN, RT) of **PTP, PTP+TBAF,** and **PTP+TBAF+Cd^{2+}** showing the expansion of aromatic region from 6.40 to 9.50 *ppm*.

PTP+F+Chromium

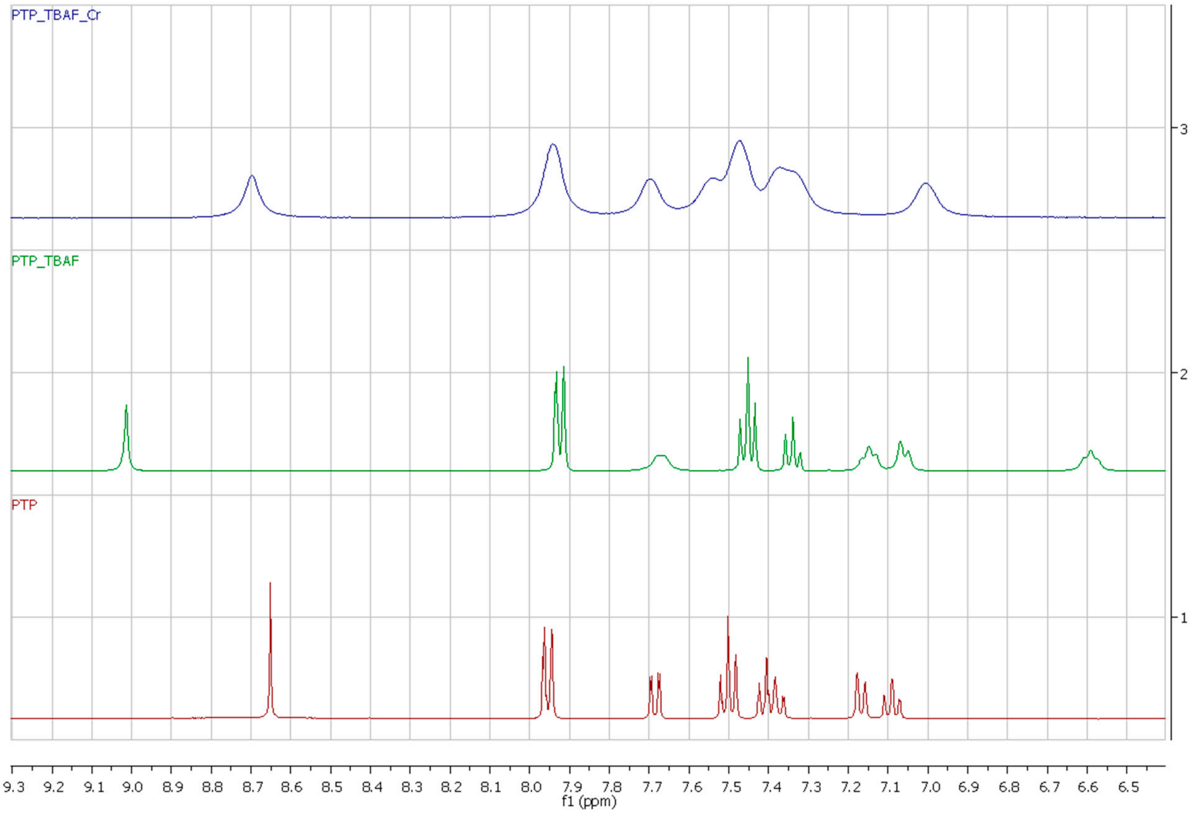

Figure S8: Stacked ^{1}H-NMR spectra (^{1}H = 400 MHz, CD_3CN, RT) of **PTP, PTP+TBAF,** and **PTP+TBAF+Cr^{3+}** showing the expansion of aromatic region from 6.40 to 9.30 *ppm*.

PTP+F+Iron (III)

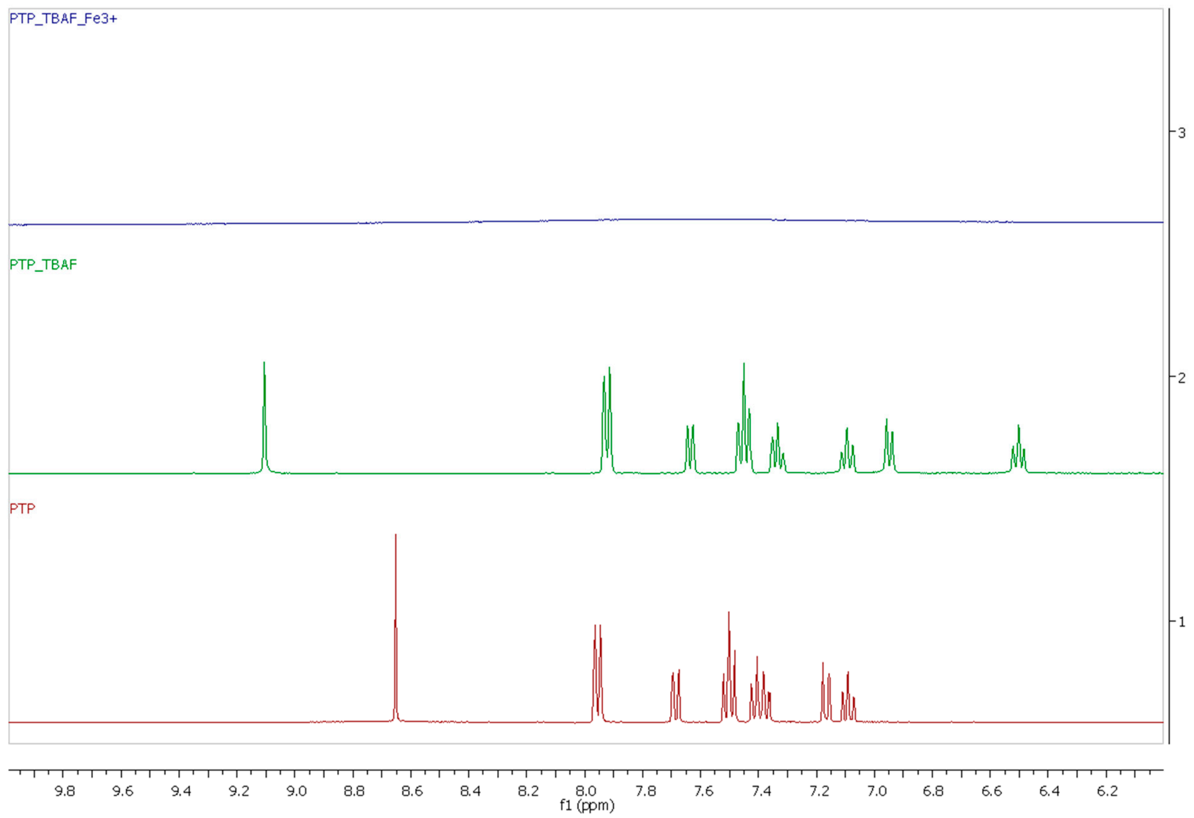

Figure S9: Stacked ^{1}H-NMR spectra (^{1}H = 400 MHz, CD_3CN, RT) of **PTP, PTP+TBAF,** and **PTP+TBAF+Fe^{3+}** showing the expansion of aromatic region from 6.10 to 10.00 *ppm*.

PTP+F+Iron (II)

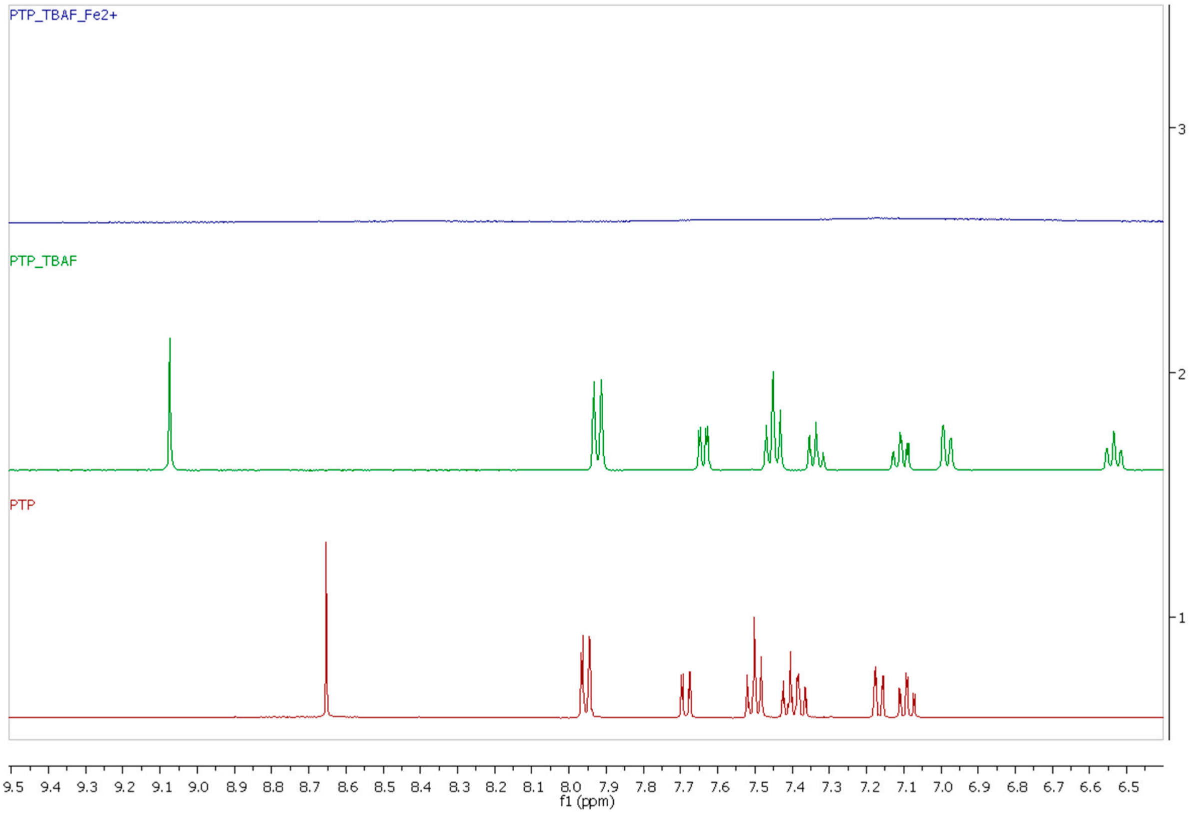

Figure S10: Stacked ^{1}H-NMR spectra (^{1}H = 400 MHz, CD_3CN, RT) of **PTP, PTP+TBAF,** and **PTP+TBAF+Fe^{2+}** showing the expansion of aromatic region from 6.40 to 9.50 *ppm*.

Benesi-Hildebrand (B-H) plots and calculation of equilibrium constants for the PTP-Fluoride-metals combination

Figs. (a) and (b) Zn^{2+} system: 1/ (F – F_0) was graphed against 1/[Zn^{2+}] using equation 1, which yielded a linear plot, and the binding constant was determined from the slope.

$$1/(F - F_0) = 1/(F' - F_0) + 1/K_b.\ 1/(F' - F_0).\ 1/[Zn^{2+}] \ .... (1)$$

Where F_0, F, and F' are the fluorescence intensities of **PTP** at 430 nm in the absence, at an intermediate concentration, and at a concentration of complete saturation of **PTP** with Zn^{2+}, respectively.

The equilibrium constant (K_{eq}) was calculated from the inverse slope of the linear plot of (F – F_0)/ (F' – F) vs [Zn^{2+}].

Figs. (c) and (d) Cu^{2+} system: 1/ (A – A_0) was graphed against 1/[Cu^{2+}] using equation 2, which yielded a linear plot, and the binding constant was determined from the slope.

$$1/(A - A_0) = 1/(A' - A_0) + 1/K_b.\ 1/(A' - A_0).\ 1/[Cu^{2+}] \ .... (2)$$

where A_0, A, and A' are the absorbances of **PTP** at 345 nm in the absence, at an intermediate concentration, and at a concentration of complete saturation of **PTP** with Cu^{2+}, respectively.

The equilibrium constant (K_{eq}) was calculated from the inverse slope of the linear plot of (A – A_0)/ (A' – A) vs [Cu^{2+}].

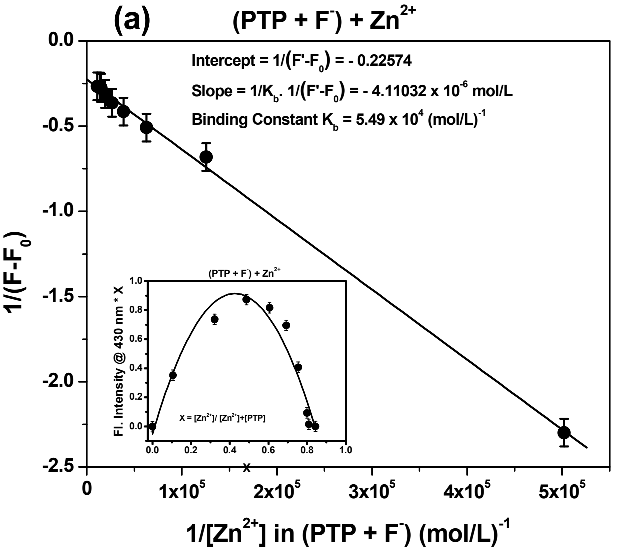

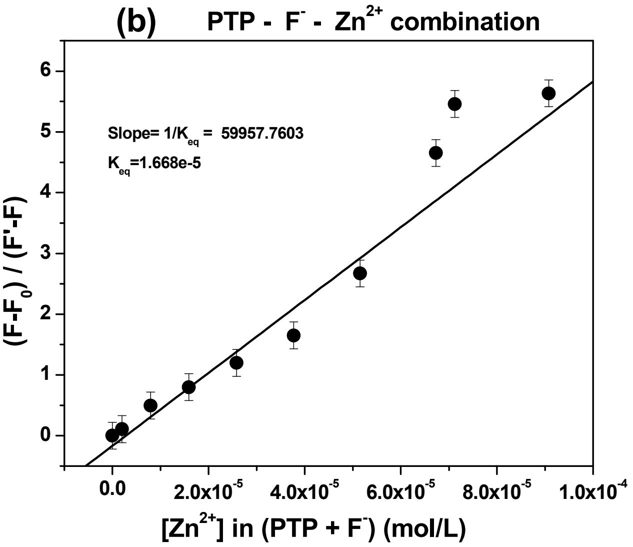

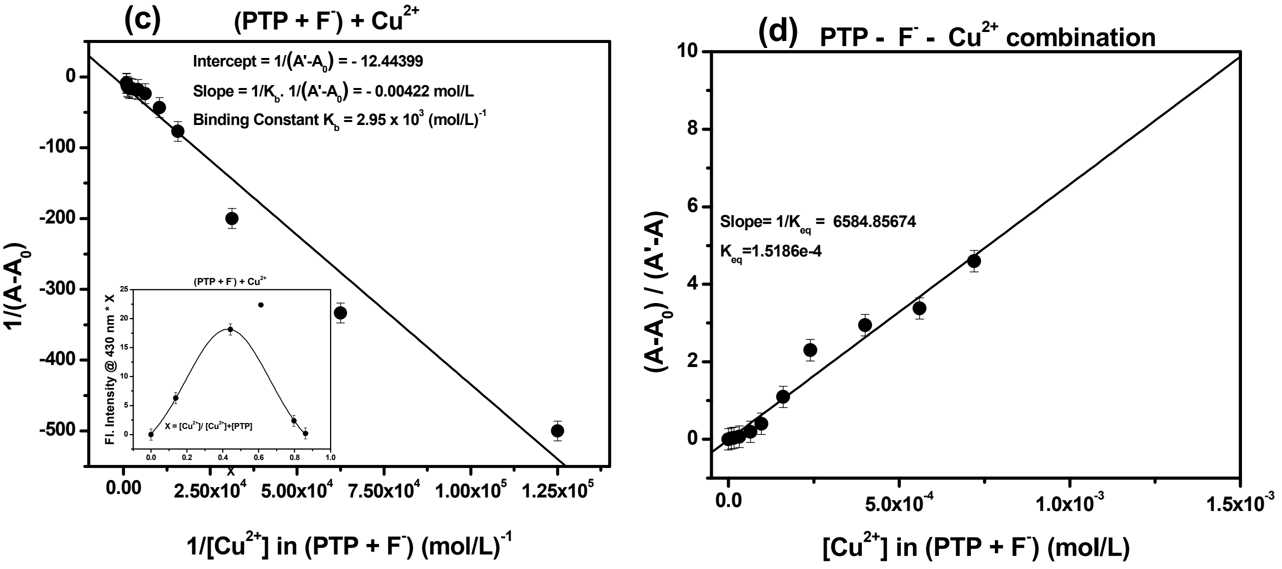

Figure S11. (a) and (c) represent Benesi-Hildebrand plots of **PTP** with the addition of Zinc and Copper perchlorate salts, respectively, in the **PTP**–fluoride complex, showing a linear relationship. (b) and (d) represent linear plots for the calculation of equilibrium constants (K_{eq}) for the PTP–fluoride Zinc and Copper complexes, respectively. The insets of Figs. (a) and (c) depict Job's plot of Zn^{2+} and Cu^{2+}, respectively, in **PTP**–fluoride medium using fluorescence and absorbance data. For Zinc (II), the fluorescence decrease was monitored at 430 nm, whereas that for Copper (II) absorbance was monitored at 345 nm. The fluorescence or the absorbance was graphed against the mole fraction, [**PTP**]/[**PTP**+metals]. Both of them yielded a peak maximum of ~0.45, indicating a 1:1 interaction.

Single Crystal X-ray spectroscopic study - PTP

Table S1: Crystal data and structure refinement for PTP.

Identification code	PTP
Empirical formula	$C_{14}H_{11}N_3O$
Formula weight	237.26
Temperature/K	99.99(10)
Crystal system	monoclinic
Space group	Pn
a/Å	5.3970(3)
b/Å	5.2119(3)
c/Å	19.8612(13)
α/°	90
β/°	95.169(6)
γ/°	90
Volume/Å^3	556.40(6)
Z	2
ρ_{calc}g/cm^3	1.416
μ/mm^{-1}	0.751
F(000)	248.0
Crystal size/mm^3	0.1 × 0.05 × 0.04
Radiation	Cu Kα (λ = 1.54184)
2Θ range for data collection/°	8.942 to 136.398
Index ranges	$-6 \leq h \leq 5$, $-6 \leq k \leq 6$, $-23 \leq l \leq 23$
Reflections collected	4233
Independent reflections	1421 [R_{int} = 0.0275, R_{sigma} = 0.0231]
Data/restraints/parameters	1421/2/165
Goodness-of-fit on F^2	1.265
Final R indexes [I>=2σ (I)]	R_1 = 0.0926, wR_2 = 0.2539
Final R indexes [all data]	R_1 = 0.0935, wR_2 = 0.2557
Largest diff. peak/hole / e Å^{-3}	0.72/-0.51
Flack parameter	1.3(11)

Table S2: Fractional Atomic Coordinates (×10[4]) and Equivalent Isotropic Displacement Parameters (Å[2]×10[3]) for PTP. U_{eq} is defined as 1/3 of the trace of the orthogonalised U_{IJ} tensor.

Atom	*x*	*y*	*z*	U(eq)
O1	-3358(10)	-397(9)	6782(3)	47.6(12)
N1	74(11)	3648(10)	6979(3)	45.2(13)
N2	1458(11)	5832(11)	7076(3)	48.9(15)
N3	2967(12)	5900(11)	6594(4)	51.9(16)
C1	2565(12)	3819(12)	6171(3)	41.4(15)
C2	697(15)	2373(12)	6439(4)	47.8(16)
C3	-1700(12)	3074(12)	7450(4)	43.7(15)
C4	-3382(14)	1073(12)	7338(3)	45.7(16)
C5	-5142(13)	699(11)	7807(4)	43.1(15)
C6	-5191(13)	2322(13)	8372(4)	46.7(16)
C7	-3502(13)	4208(13)	8481(4)	44.7(16)
C8	-1728(13)	4618(12)	8025(4)	45.4(15)
C9	4020(12)	3399(12)	5585(4)	43.8(15)
C10	5962(13)	5009(13)	5461(4)	47.0(16)
C11	7304(15)	4592(14)	4927(4)	49.4(16)
C12	6777(13)	2572(13)	4491(4)	47.7(16)
C13	4791(14)	947(13)	4598(4)	48.7(16)
C14	3412(13)	1365(13)	5158(4)	46.2(15)

Table S3: Anisotropic Displacement Parameters (Å[2]×10[3]) for PTP. The Anisotropic displacement factor exponent takes the form: $-2\pi^2[h^2a^{*2}U_{11}+2hka^*b^*U_{12}+\ldots]$.

Atom	U_{11}	U_{22}	U_{33}	U_{23}	U_{13}	U_{12}
O1	44(2)	37(2)	64(3)	-3(2)	15(2)	-12.8(18)
N1	46(3)	31(3)	60(3)	-1(2)	12(3)	-7(2)
N2	46(3)	38(3)	65(4)	-5(2)	17(3)	-8(2)
N3	52(4)	38(3)	67(4)	3(3)	17(3)	-10(2)
C1	38(3)	41(3)	48(4)	5(3)	19(3)	-1(3)
C2	46(3)	36(3)	63(4)	1(3)	11(3)	-11(3)
C3	40(3)	32(3)	61(4)	5(3)	12(3)	3(3)
C4	53(4)	36(3)	48(3)	4(3)	8(3)	13(3)
C5	50(4)	26(3)	54(4)	-3(3)	13(3)	-6(3)
C6	48(4)	38(3)	57(4)	2(3)	18(3)	6(3)
C7	44(4)	34(3)	58(4)	-5(3)	14(3)	1(2)
C8	40(3)	33(3)	65(4)	-1(3)	14(3)	-7(3)
C9	42(4)	33(3)	58(4)	-3(3)	10(3)	4(3)

Table S3: Anisotropic Displacement Parameters ($Å^2×10^3$) for PTP. The Anisotropic displacement factor exponent takes the form: $-2\pi^2[h^2a^{*2}U_{11}+2hka^*b^*U_{12}+…]$.

Atom	U_{11}	U_{22}	U_{33}	U_{23}	U_{13}	U_{12}
C10	43(3)	37(3)	63(4)	-3(3)	15(3)	0(3)
C11	49(4)	39(3)	62(4)	2(3)	14(3)	-1(3)
C12	43(4)	47(4)	55(4)	2(3)	15(3)	5(3)
C13	47(4)	35(3)	66(4)	-4(3)	19(3)	4(3)
C14	47(4)	37(3)	57(4)	0(3)	15(3)	-5(3)

Table S4: Bond Lengths for PTP.

Atom	Atom	Length/Å	Atom	Atom	Length/Å
O1	C4	1.344(9)	C4	C5	1.403(10)
N1	N2	1.365(8)	C5	C6	1.407(10)
N1	C2	1.331(10)	C6	C7	1.345(11)
N1	C3	1.428(9)	C7	C8	1.392(10)
N2	N3	1.313(9)	C9	C10	1.382(10)
N3	C1	1.376(10)	C9	C14	1.379(10)
C1	C2	1.401(9)	C10	C11	1.355(11)
C1	C9	1.479(9)	C11	C12	1.377(11)
C3	C4	1.388(10)	C12	C13	1.397(11)
C3	C8	1.399(10)	C13	C14	1.409(10)

Table S5: Bond Angles for PTP.

Atom	Atom	Atom	Angle/°	Atom	Atom	Atom	Angle/°
N2	N1	C3	118.2(6)	C3	C4	C5	118.1(6)
C2	N1	N2	110.8(6)	C4	C5	C6	120.6(6)
C2	N1	C3	131.0(6)	C7	C6	C5	120.4(7)
N3	N2	N1	106.7(6)	C6	C7	C8	120.3(6)
N2	N3	C1	110.5(6)	C7	C8	C3	120.1(6)
N3	C1	C2	105.7(6)	C10	C9	C1	121.0(6)
N3	C1	C9	122.1(6)	C14	C9	C1	119.0(6)
C2	C1	C9	132.2(7)	C14	C9	C10	120.0(7)
N1	C2	C1	106.3(6)	C11	C10	C9	120.6(7)
C4	C3	N1	121.4(6)	C10	C11	C12	121.4(7)
C4	C3	C8	120.5(6)	C11	C12	C13	119.1(7)
C8	C3	N1	118.1(6)	C12	C13	C14	119.5(7)
O1	C4	C3	120.3(7)	C9	C14	C13	119.4(6)
O1	C4	C5	121.6(6)				

Table S6: Hydrogen Bonds for PTP.

D	H	A	d(D-H)/Å	d(H-A)/Å	d(D-A)/Å	D-H-A/°
O1	H1	N3[1]	0.84	1.97	2.769(8)	157.5

[1]-1+X,-1+Y,+Z

Table S7: Torsion Angles for PTP.

A	B	C	D	Angle/˚	A	B	C	D	Angle/˚
O1	C4	C5	C6	-177.6(6)	C2	C1	C9	C10	-173.5(8)
N1	N2	N3	C1	1.2(8)	C2	C1	C9	C14	6.6(11)
N1	C3	C4	O1	0.7(10)	C3	N1	N2	N3	178.6(6)
N1	C3	C4	C5	-177.2(6)	C3	N1	C2	C1	-179.4(7)
N1	C3	C8	C7	176.9(7)	C3	C4	C5	C6	0.2(9)
N2	N1	C2	C1	-0.5(8)	C4	C3	C8	C7	-2.1(10)
N2	N1	C3	C4	171.8(6)	C4	C5	C6	C7	-2.0(10)
N2	N1	C3	C8	-7.1(9)	C5	C6	C7	C8	1.7(11)
N2	N3	C1	C2	-1.5(8)	C6	C7	C8	C3	0.3(11)
N2	N3	C1	C9	-179.6(6)	C8	C3	C4	O1	179.6(6)
N3	C1	C2	N1	1.1(8)	C8	C3	C4	C5	1.8(9)
N3	C1	C9	C10	4.1(10)	C9	C1	C2	N1	179.0(7)
N3	C1	C9	C14	-175.8(7)	C9	C10	C11	C12	0.3(11)
C1	C9	C10	C11	179.2(7)	C10	C9	C14	C13	0.2(10)
C1	C9	C14	C13	-179.9(7)	C10	C11	C12	C13	1.0(11)
C2	N1	N2	N3	-0.4(8)	C11	C12	C13	C14	-1.7(11)
C2	N1	C3	C4	-9.3(11)	C12	C13	C14	C9	1.1(11)
C2	N1	C3	C8	171.8(7)	C14	C9	C10	C11	-0.9(10)

Table S8: Hydrogen Atom Coordinates (Å×10^4) and Isotropic Displacement Parameters (Å^2×10^3) for PTP.

Atom	*x*	*y*	*z*	U(eq)
H1	-4726.26	-1162.76	6712.28	71
H2	12.18	797.7	6269.37	57
H5	-6310.48	-661.25	7742.8	52
H6	-6425.27	2083.06	8678.64	56
H7	-3519.77	5264.77	8870.08	54
H8	-534.47	5948.49	8105.23	54
H10	6359.78	6421.79	5753.17	56
H11	8636.88	5717.07	4851.9	59

Table S8: Hydrogen Atom Coordinates (Å×10^4) and Isotropic Displacement Parameters (Å^2×10^3) for PTP.

Atom	*x*	*y*	*z*	U(eq)
H12	7750.91	2286.05	4122.87	57
H13	4372.7	-430.02	4295.27	58
H14	2075.19	255.72	5239.75	55

Experimental

Single crystals of PTP were collected through slow evaporation of dichloromethane. A suitable crystal was selected and mounted on a **Rigaku XtaLAB Synergy-i XRD** diffractometer and processed using CrysAlis Pro (Rigaku) software [**2**]. The crystal was kept at 100 K during data collection. Using Olex2 [**3**], the structure was solved with the SHELXT [**4**] structure solution program using Intrinsic Phasing and refined with the SHELXL [**5**] refinement package using Least Squares minimization.

2. Rigaku. O.D. CrysAlis Pro, Oxford Diffraction/Agilent Technologies UK Ltd: Yarnton, England (2018)
3. Dolomanov, O.V., Bourhis, L.J., Gildea, R.J, Howard, J.A.K. & Puschmann, H. (2009), J. Appl. Cryst. 42, 339-341.
4. Sheldrick, G.M. (2015). Acta Cryst. A71, 3-8.
5. Sheldrick, G.M. (2015). Acta Cryst. C71, 3-8.

This report was created with Olex2, compiled on 2023.07.18 svn.rf166f9f3 for OlexSys. Please let us know if there are any errors or if you would like to have additional features.
